# From farm to microbe: organic amendments and soil texture as drivers of soil Microbiome composition

**DOI:** 10.1186/s40793-025-00815-1

**Published:** 2025-12-29

**Authors:** Maya Subberwal, Madeline Giles, Roy Neilson, David Roberts, Sandra Caul, Susan Mitchell, Umer Zeeshan Ijaz

**Affiliations:** 1 James Watt School of Engineering, University of Glasgow, Glasgow, G12 8QQ UK; 2 The James Hutton Institute, Invergowrie, Dundee, DD2 5DA UK

**Keywords:** 16S rRNA, Core microbiome, Organic amendments, Soil microbial diversity, Soil texture

## Abstract

**Background:**

Understanding how agricultural practices affect soil bacterial communities is vital to mitigating the negative impacts of intensive agriculture on soil health and preventing further deterioration of arable land. Increasing pressure on agricultural land necessitates careful management of our productive soil. This study investigates the interaction between organic amendments and soil texture in agricultural soils (n = 93) used for arable production, using a 16S rRNA-sequencing based microbial community analysis. Amendments include slurry, digestate, and farmyard manure. Additionally, soil physicochemical parameters were collected to explore the drivers of patterns of soil microbial diversity.

**Results:**

Microbial community composition was significantly influenced by organic amendments and soil texture, which both exerted distinct selective pressures. Analysis using 16S rRNA-sequencing and advanced modelling identified significant factors affecting community structure, including soil calcium levels, the crop grown one year previously, loss on ignition (LOI), and farm ID. The genus *Candidatus Nitrosotalea* was found to be positively associated with application of farmyard manure, while genus *AD3* (phyla Chloroflexi) was found to be negatively associated with application of digestate and slurry.

**Conclusions:**

The results highlight the importance of considering multiple, interacting factors when trying to establish how agricultural practice affects soil microbial communities. Our findings underscore the need for tailored management strategies – specific to the local environment and available resources – to promote soil health.

**Supplementary Information:**

The online version contains supplementary material available at 10.1186/s40793-025-00815-1.

## Introduction

Globally, climate change and population growth are increasing the pressure on agricultural systems to produce more food in a more sustainable way. Central to achieving this is ensuring soil health, a term described by the EU Mission Soil as “*the continued capacity of soils to support ecosystem services”* [1]. The ability of soils to provide ecosystem services is often underpinned by diverse microbial communities that are key mediators of a broad range of processes, including plant growth promotion, pathogen suppression, nutrient cycling, and soil organic matter stabilisation [2]. The assembly, formation and composition of microbial communities are strongly influenced by environmental conditions [3–5] and consequently, can be altered by agricultural practices, potentially leading to reduced soil fertility and declining ecosystem resilience [6]. It is therefore crucial to examine how variable abiotic factors within agricultural soils, such as pH and soil texture, shape microbial community structure [7–9]. We must understand how agricultural practices, e.g., the application of organic amendments, impact these communities and furthermore, we need to determine how these practices interact with fixed soil properties to shape microbial communities. This understanding is vital for predicting microbial community responses in agroecosystems and supporting agronomic interventions for soil health.

Current understanding suggests that agricultural practices can select for microbes adapted to the conditions these practices create [10, 11], altering the native microbial community structure [12]. Altered microbial communities can directly influence plant metabolism and development [13, 14], as well as broader soil functions such as C and N cycling [15]. Conventional agriculture can contribute to dysbiosis within soil communities, for example inversion tillage increases fragmentation in microbial communities and decreases nutrient availability [16] and excessive application of fertiliser can contribute to dysbiosis [17]. To address the challenges presented by conventional intensive agriculture, organic soil amendments are applied because of their inherent biological and chemical properties that lead to improved soil quality and enhanced microbial activity. Organic amendments confer numerous benefits including high organic matter content, high nutrient content, and readily accessible, highly diverse microbial populations [18, 19]. Organic amendments vary in properties based on source material, with factors such as dry matter content and C to N ratios varying both within and between amendment types. Application of organic amendments changes physicochemical conditions in the soil, creating new niches and selective pressures, potentially affecting microbial community composition and function [20]. Consequently, application of organic amendments has been shown to have a positive effect on the soil microbiome, increasing functional resilience and community stability, which is believed to contribute to healthier soil systems [21]. However, the impact of organic amendments has the potential to vary between soil textures with different soils impacting the retention of water, availability of nutrients and underlying microbial community structure. This makes it important to understand how the interaction of organic amendments and soil textures can affect the underlying ecological processes structuring microbial communities in soils.

Understanding how microbial communities are structured can help us better support soil health and sustainable agricultural practices. By integrating broad-scale patterns from microbial ecology with macroecology, we can disentangle the relative contributions of neutral processes (stochastic events) and environmental filtering (deterministic factors) in shaping microbial community structure [22]. Neutral processes (e.g., random birth and death events, dispersal limitations) contribute to stochastic fluctuations in microbial populations, shaping microbial communities over time. Conversely, environmental filtering involves deterministic selective pressures that are exerted by environmental factors, influencing communities according to abiotic conditions. In the present context, this means the physicochemical properties of different soil textures will interact variably with the biological and physicochemical properties of organic amendments. While the direct influence of soil texture on microbial composition is recognised [23–25], the interactions between fixed factors such as soil texture and variable factors such as organic amendments in shaping microbial communities remain poorly understood [26]. Understanding how agricultural practices interact with the environment to define microbial community composition is crucial for determining the universality of the structuring mechanisms and for predicting the long-term effects of amendment strategies.

Our research therefore aims to investigate the impact of three organic soil amendments on the diversity of microbial communities associated with four different soil textures. The objectives of this study were to (1) identify if a core microbial community is maintained given variable soil texture and the studied organic soil amendments, and (2) determine if soil texture, acting as an environmental filter, has a role in shaping the microbiome structure in the context of organic soil amendment application. By investigating the interplay between soil texture and organic amendments on microbial community assembly, this study aims to contribute to the development of more sustainable and resilient agricultural practices.

## Materials and methods

### Soil sampling and sample selection

Soil samples used in this study were an archived subset of samples collected from agricultural soils across the UK as part of a study focussed on understanding the drivers of soil nematode communities (Technology Strategy Board TS/M012956/1). To minimise any potential effect of biogeography and crop, samples selected for use in this study were collected from farms across North-Eastern Scotland (*n* = 14), and from fields sown with winter wheat (most sampled crop type), representing a total of 116 samples, comprising four soil textures (Sandy Silt Loam, Sandy Loam, Silty Clay Loam, and Clay Loam). For sampling, fields were divided into six approximately equal-sized areas. Within each area, a random 20 m by 20 m area was selected to sample. Approximately thirty samples were collected along a W pattern [27] to a depth of 10 cm using a grass plot sampler (Van Walt, Haslemere, UK) to generate a composite sample per area. In-field, samples were stored in cool boxes (~ 4 °C) and transported to the laboratory. On arrival at the laboratory, 10 g of fresh soil was removed from the composite sample and stored at − 80 °C until DNA processing. A further subsample of each sample was subject to soil physiochemical analysis (SAC Consulting, UK; Supplementary Materials Table S3). A detailed list of all metadata collected is outlined in Table S1-4. Detailed information concerning specific farm locations for the chosen sites is subject to GDPR and therefore unavailable. For this study, the organic amendments applied to soil were described as follows: (None, Farmyard Manure (FYM), Digestate, or Slurry). Although not known, it is assumed that all digestate was produced on-farm using manure and crop materials as a primary feedstock.

### Soil physiochemical analysis

A further subsample of each sample was subject to soil physiochemical analysis (SAC Consulting, UK; Supplementary Materials Table S3). The method for LOI determination is as follows: samples were weighed, dehydrated in a Gallenkamp Hotbox oven at 100 °C for 3 h and then cooled in a desiccator for 1 h. Samples were ashed in a Pyro Advanced Microwave Muffle Furnace at 550 °C for 2 h 15 min before being cooled in a desiccator for 1 h. LOI values were calculated automatically through a four-place electronic balance by the direct data capture software Labware LIMS. A routine extraction for the major elements (P, K, Mg, Ca, Na) was conducted through dissolution by weak acid, as follows. Samples were mixed thoroughly, 10 ml of soil was mixed with 50 ml of a weak acid extractant solution (ammonium acetate/acetic acid) and mixed in a Gallenkamp Orbital Shaker for 30 min. Samples were then filtered through No. 40 filter paper into 8 ml tubes and analysed using a PerkinElmer Optima 4300 DV ICP-OES system, controlled via Winlab32 software. Quality control was maintained by running ICP-OES calibration standards and quality control solutions in addition to an SAC in-house check soil with every batch.

### DNA extraction and 16SrRNA library preparation

DNA was extracted using a Qiagen DNAeasy PowerSoil Kit [28] according to the manufacturer’s instructions.

16S rRNA gene amplicon libraries of the V4 region were sequenced at the James Hutton Institute, using a 2 × 300 bp run on an Illumina MiSeq. For library creation, samples were initially amplified using 515F (GTGYCAGCMGCCGCGGTAA) and 806R (GGACTACNVGGGTWTCTAAT) primers [29, 30], KAPA HiFi Hot-Start Taq (Roche, USA) and 2.5 μl of DNA template. The PCR conditions were as follows: 95 °C for 3 min then 25 cycles of 95 °C for 20 s, 55 °C for 10 s, 72 °C for 20 s, before a final extension step of 72 °C for 5 min. PCR products were cleaned up using Agencourt AMPure beads (Beckman Coulter, Indianapolis, U.S.A.) as per the standard Illumina protocol. Nextera XT indexes (Illumina, San Diego, U.S.A.) were added following the manufacturer’s instructions, and the resulting PCR product was further cleaned up using AmpPure beads. PCR negative controls and blank DNA extractions up to the pooling stage were carried out.

All samples were quantified using picogreen and pooled to equimolar amounts. Samples were quality controlled using a 2100 Bioanalyzer (Agilent, Santa Clara, U.S.A.), before being run on an Illumina MiSeq System (Illumina, San Diego, U.S.A.).

### Bioinformatics

From 222 samples, we obtained 5,545,798 paired end reads. VSEARCH v2.3.4 [31] was used to cluster similar sequences into operational taxonomic units (OTUs), with a 99% sequence identity similarity threshold [32]. After excluding singletons, resulting in the exclusion of a single sample, this yielded 99,932 OTUs from 221 samples. Representative OTUs were recovered at 99% similarity using the same approach as referenced above.

We used PiCRUST2 within the QIIME v2019.7 environment [33] to recover KEGG enzymes (10,543 enzymes for 221 samples) and MetaCyc pathway predictions (489 pathways for 221 samples) [34, 35]. QIIME2 was used to generate a BIOM file containing a final OTU abundance table of *n* = 221 × P = 99,932. Summary statistics of OTUs per sample were as follows: [Min:3; 1st quartile: 13,506; median: 19,146; mean: 17,568; 3rd quartile: 22,448; max: 42,259]. DADA2 was also used to generate a BIOM file [36], yielding a final ASV abundance table of *n* = 222 × P = 37,032 (without the exclusion of the singleton), with summary statistics of ASVs per sample as: [Min = 0; 1st quartile: 5,465; median: 8,347; mean: 8,715; 3rd quartile: 11,956; max: 25,206].

While ASVs offer higher theoretical resolution, we found that the use of distance-based OTUs (99% clustering) provides a more biologically relevant and stable ecological signal for our specific study objectives. First, ASV methods risk biologically meaningless over-splitting of single genomes, which artificially inflates diversity and can lead to erroneous ecological conclusions [37]. OTU clustering provides greater robustness to uncorrected sequencing noise, mitigating the impact of residual sequencing errors and additionally, the removal of singletons reduces spurious ecological variation [38]. Finally, OTUs better retain the rare biosphere in complex environments [39]. For these reasons, to ensure accurate interpretation of the biosphere and due to the higher number of features and therefore improved statistical power from the VSEARCH pipeline, OTUs were used for this study.

### Statistical analysis

**Fig. 1:**
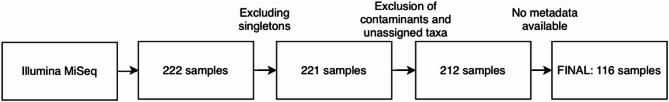
Schematic representing the number of samples during each bioinformatic stage from initial sequencing to final analysis

During pre-processing, we selected samples with > 5000 reads, removed contaminants such as mitochondria and chloroplasts, as well as OTUs that were unassigned at any level. This led to the exclusion of 9 samples, resulting in a final table of *n* = 212 × P = 96,937 OTUs with summary statistics as follows: [Min:5,079; 1st quartile: 14,230; median: 19,361; mean: 18,110; 3rd quartile: 22,296; max: 41,827]. Due to gaps in the metadata associated with the soil archive, 96 samples had no metadata and therefore were removed from the dataset giving a total of 116 samples. A schematic to represent the sample counts at each stage can be seen in Fig. 1.

The following statistical analysis was conducted in R v4.4.1. The R package, vegan v2.6.10 [40] was used for alpha and beta diversity analyses. To calculate alpha diversity (after rarefying to minimum library size) we used: (i) Shannon entropy and (ii) Chao1 richness. Pair-wise analysis of variance (ANOVA) p-values were calculated using R’s aov() function. PiCRUST2 predictions were used to estimate community functional dissimilarity. PiCRUST2 predictions may not perfectly reflect actual functional potential of the microbial community though predictions can be useful indicators of community functional diversity. Continuous environmental variables were visualised to determine differences between primary conditions (see Fig. S1).

Principal Coordinate Analysis (PCoA) was used to visualise differences in the abundance table with three different distance measures: (i) Bray–Curtis distance, to visualise compositional changes; (ii) Unweighted UniFrac Distance, to identify phylogenetic changes between samples [41] and (iii) Hierarchical Meta-Storms (HMS), to give a weighted dissimilarity measure of differences in functional beta diversity, calculated from upward hierarchical propagation of observed KEGG orthologs [42]. PERMANOVA analyses using vegan determined different sources of variability within community structures. Beta diversity was visually assessed using beta contour plots and null models. Phylogenetic distance and community functional distance matrices were combined with a regression model to indicate the stability of communities to external perturbations. Additionally, the cca function within vegan was used to conduct a canonical correspondence analysis to explore the relationships between environmental variables.

To understand the influence of the environment on microbial community assemblage and to explore phylogenetic dispersion in the data, beta Nearest Taxon Index (βNTI) was calculated using the picante v1.8.2 package [43]. βNTI measures local divergences in phylogeny, putting more emphasis on terminal clades. Positive values of βNTI indicate co-occurrence of phylogenetically similar species, more frequently than expected by chance. Negative values indicate phylogenetic overdispersion.

Redundancy analysis was employed to select the most informative model of key drivers of microbial assembly by comparing the amount of variation explained by different combinations of environmental factors. To select parameters most strongly associated with the observed variance between communities, we used vegan’s capscale() and ordistep() functions to apply redundancy analysis (RDA) on different beta diversity distances as a variable selection approach before applying PERMANOVA analysis [44]. The following variables were input into the PERMANOVA analyses: Farm ID, Soil Texture, Crop Year 4, Crop Year 3, Crop Year 2, Crop Year 1, Rotation Type, Straw Incorporation, Main Soil Amendment, Second Soil Amendment, Cultivation Method, Nematicide Applied, Soil pH, Soil Phosphorus (P), Soil Potassium (K), Soil Magnesium (Mg), Soil Calcium (Ca), Soil Sodium (Na), and Soil (LOI).

To determine which environmental conditions favoured specific microbial taxa through environmental filtering, we performed a differential abundance analysis using raw amplicon count data. We used the DESeqDataSetFromMatrix() function from the DESeq2 v1.46.0 package [45] on the raw counts with the following settings: adjusted p-value significance cut-off of 0.05; log2 fold change cut-off of 2. DESeq2 was selected because it internally models library size and compositional effects using a robust Generalised Linear Model (GLM). Differentially abundant taxa were visualised using Total Sum Scaling (TSS) followed by Centralised Log Ratio (CLR) transformation on raw abundance values. TSS is a normalisation technique that standardises values, CLR is a normalisation and transformation technique that uses a log-ratio transformation to remove compositional bias [46].

A Generalised Linear Latent Variable Model (GLLVM) was used to investigate the relationships between sources of variability and individual taxa [47]. This method explicitly accounts for unobserved factors that may influence microbial community structure. The GLLVM extends standard generalised linear models by incorporating latent variables. Specifically, the model regresses the mean abundance ${\mu }_{ij}$ (for *i*-th sample and *j*-th microbe) of individual microbes against covariates ${x}_{i}$, through the incorporation of latent variables ${u}_{i}$ as shown in Eq. (1):

1 $$ g\left( {\mu_{ij} } \right) = \eta_{ij} = \alpha_{i} + \beta_{0j} + x_{i}^{T} \beta_{j} + u_{i}^{T} \theta_{j} $$

where ${\beta }_{j}$ are microbe-specific coefficients associated with individual covariates (with a 95% confidence interval, not crossing the 0-boundary giving directionality), and ${\theta }_{j}$ are the coefficients associated with the latent variables. ${\beta }_{0j}$ are microbe-specific intercepts, whilst ${\alpha }_{i}$ are optional sample effects, chosen either as fixed or random effects. This approach allows for the simultaneous modelling of observed and unobserved influences on microbial community composition.

The GLLVM was generated using the following covariates: Farm ID (KB; KN; GW; HF; WB; EH; AG; EN; BC; ER; DB; MB; IB; FF [REF]); Crop Year 2 (Spring Peas (SPEAS); Seed Potato (SPOT); Winter Oilseed Rape (WOSR); Ware Potato (WPOT); Winter Wheat (WW); Fallow [REF]); Main Soil Amendment (FYM; Digestate; Slurry; None [REF]):; Soil LOI (continuous); and Soil Ca (continuous). Here [REF] refers to the reference factor, for which no beta coefficients are obtained.

The core microbiome was defined using the abundance/occupancy approach of Shade and Stopnisek, [48]. This method identifies taxa present across all samples, considering both their proportional contribution (abundance) and detection frequency (occupancy). Initial levels of occupancy were extracted from the OTU abundance table considering both site-specific occupancy and replication consistency. Taxa were then ranked using the following index:

2 $$\begin{gathered} Index_{{OTUi}} \hfill \\ \quad \quad = \frac{{\Sigma \left( {Site~specific~occupancy} \right) + \Sigma \left( {\text{Re} plication~consistency} \right)}}{2} \hfill \\ \end{gathered}$$

Bray–Curtis similarity was calculated for the whole dataset and for the top-ranking taxa. The percentage contribution of the prospective core set of taxa to beta diversity was calculated by:

3 $$ C = 1 - \frac{{BC_{core} }}{{BC_{all} }} $$

Taxa were added iteratively until the inclusion of further taxa increased the explanatory value, $C$, by less than 2%. Additionally, a neutral model was fitted to the abundance/occupancy distributions to identify the method of OTU selection.

Finally, to identify discriminant microbial signatures, CODA-LASSO was applied. Compositional data analysis (CODA) methods explicitly account for the compositional nature of microbiome data. CODA-LASSO incorporates (1) a beta-zero-sum constraint, forcing all variable coefficients to sum to zero, (2) a LASSO penalty term, inducing sparsity in the model through shrinkage of less important variables towards zero [49, 50], and (3) elastic net regularisation, a combination of Least Absolute Shrinkage and Selection Operator (LASSO) and ridge regression shrinkage, offering increased flexibility in variable selection [51]. Ridge regression shrinkage mitigates LASSO’s limitations with highly correlated features, enhancing model stability and reducing overfitting [52]. This analysis yields a subset of taxa forming a microbial signature that optimally discriminates between two variable conditions.

## Results

### Soil properties and environmental conditions

Soil calcium and LOI were found to be significant physicochemical parameters using RDA-PERMANOVA. Variation was seen in calcium levels across soil texture; silty clay loam soils had significantly higher calcium levels than either sandy silt or sandy loam type soils. No significant differences in calcium levels were found for main soil amendments (*p* > 0.05) (Supplementary Fig. S1). Loss on ignition (LOI), a proxy for soil organic matter content, was higher in silty clay loams than either sandy loam or sandy silt loam. Though sample size was low, digestate-applied soils (*n* = 4) had significantly higher values for LOI compared to soils that had farmyard manure (FYM) applied or with soils that had no amendment (Supplementary Fig. S1). Similarly, soils that had slurry applied (*n* = 4), though again a low sample size, also had significantly higher values for LOI compared to no amendment (Supplementary Fig. S1). Further detail concerning mineral analysis can be found in Supplementary Table S3.

### Microbial community structure and diversity

**Fig. 2:**
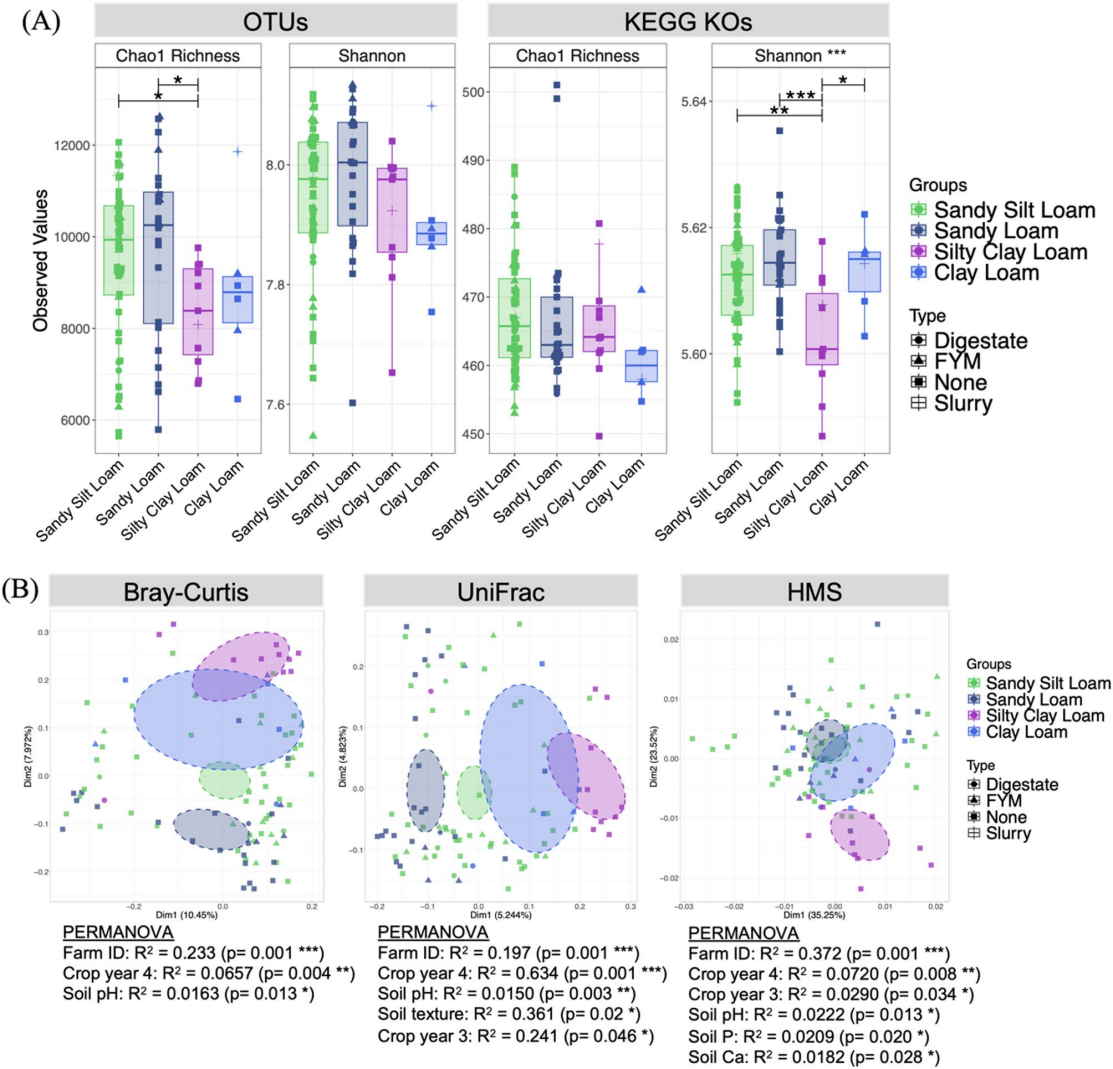
**A** boxplots representing alpha diversity for the four main soil amendments showing Chao1 measure of richness and Shannon entropy for OTUs (left panel) and KEGG orthologs (right panel). Horizontal lines at the top of the plots represent significant differences in alpha diversity between soil amendments, with asterisks denoting significance at *p < 0.05, **p < 0.01, ***p < 0.001; **B** shows PCoA plots representing beta diversity with associated PERMANOVA statistics listed below. Different symbols represent different soil textures

For alpha diversity, based on Shannon’s entropy measure, digestate-applied soils exhibited the highest level of functional richness (Fig. 2A, right panel). The alpha diversity results based on taxonomic classification were not found to be significant for any amendment application using either Shannon’s entropy measure or Chao1 richness (*p* > 0.05).

**Fig. 3:**
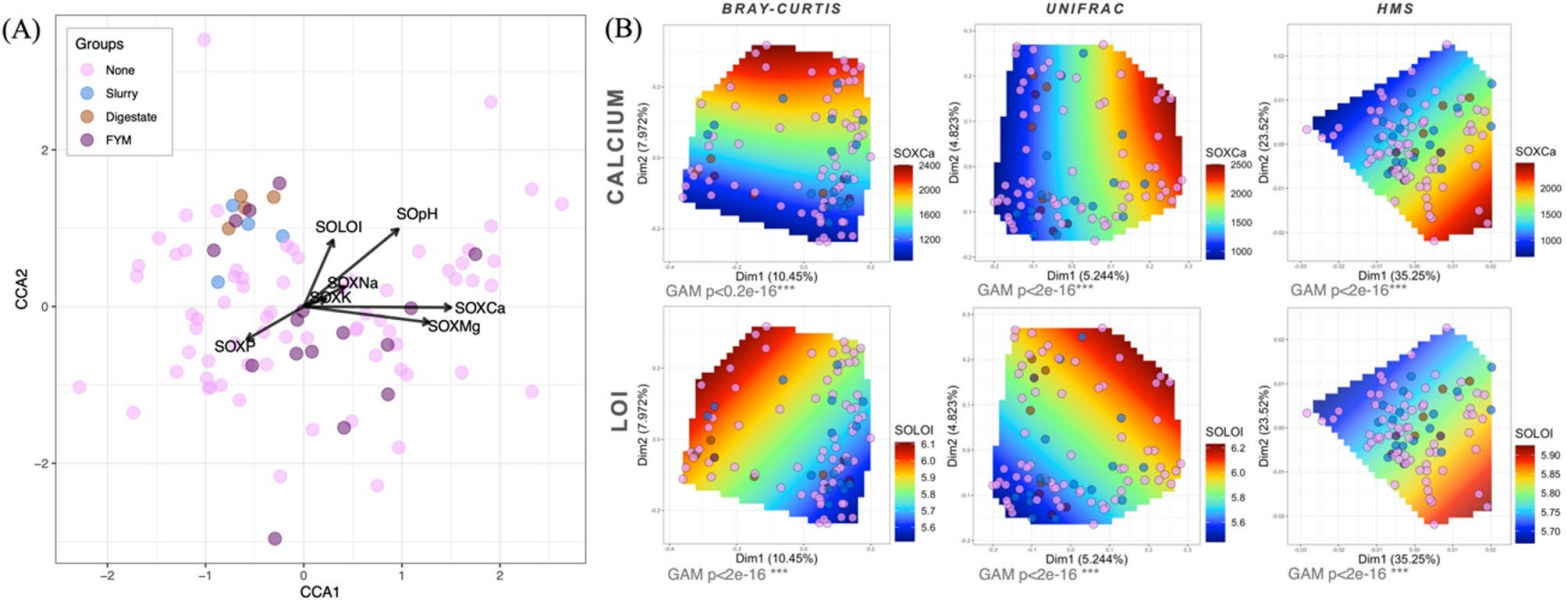
**A** Canonical Correspondence Analysis (CCA) of key covariates superimposed on the ordination of soil microbiome samples. The plot is scaled by the correlation of the vectors so that weakest predictors have shorter arrows than stronger predictors and the direction of the arrows represent the increase in the value of those covariates. **B** Smoothed surfaces of the covariates fitted onto PCoA ordination plots using penalised splines and different beta diversity distances. The method uses a generalised additive model (GAM) by regressing the covariate as C ~ S(Dim1,Dim2), where Dim1 and Dim2 are the ordination scores extracted from PCoA and S() is a spline function. Only covariates where the model fits are shown i.e., *p* < 0.05. GAM values show how well the environmental covariates fit on the ordination model, with asterisks denoting significance at ***p < 0.001

Regardless of whether distance matrices related to taxonomy (Bray–Curtis) or phylogeny (UniFrac), a similar pattern of beta diversity was observed with FYM and no amendment forming a single cluster, whereas slurry and digestate formed contiguous clusters (Fig. 2B). Results from the CCA analysis show distinct clustering of digestate and slurry applied soils towards an increased LOI (Fig. 3A). Visual inspection of the PCoA plots (Fig. 3B) identifies similar findings for both LOI and soil calcium levels, particularly evident using Bray–Curtis and UniFrac distances matrices.

Of all the parameters tested using a PERMANOVA with both forward and reverse selection (RDA) (Supplementary Table S5), LOI accounted for 1.5% variability in composition and 1.1% variability in phylogeny while calcium accounted for a slightly higher degree of variability (5.4% variability in composition; 3.6% in phylogeny, 6.4% variability in function). The crop grown the year previously accounted for 1.4% variability in composition and 1.2% variability in phylogeny while the crop grown two years previously accounted for 1.1% variability in composition. The second soil amendment (if applied) was considered significant (2.7% variability in composition, 2.0% variability in phylogeny). Similarly, combination practice (defined as the inclusion of ‘combinable-only’ biomass versus ‘combinable plus roots and potatoes’ biomass), pH, phosphorus, straw incorporation, and farm ID were all implicated as significant covariates.

### Influence of soil amendments on microbial communities

**Fig. 4:**
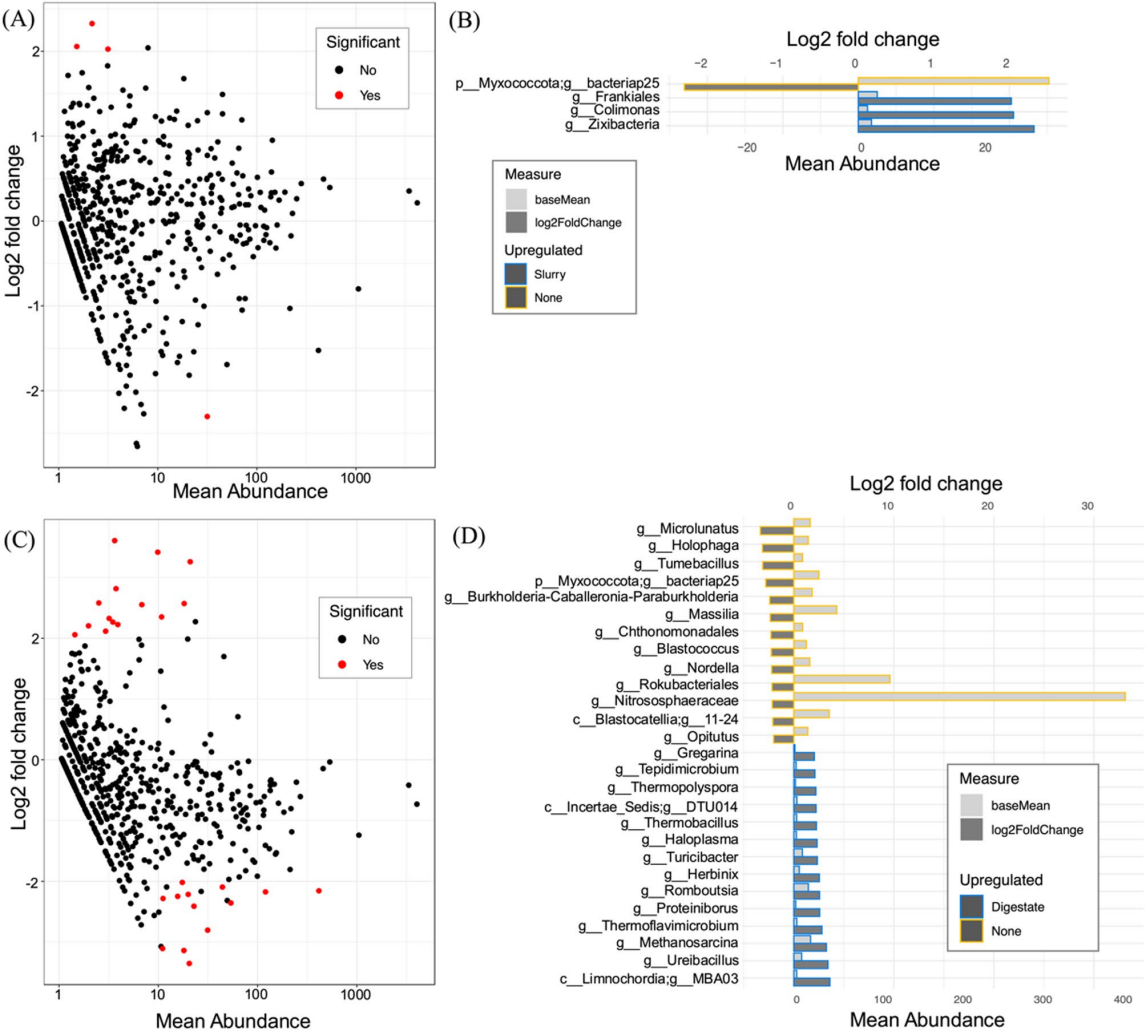
Differential abundance analysis of amendment practices using DeSEQ2. **A** and **C** represent the distribution of the mean abundance of bacterial taxa for soils with applications of slurry and digestate, respectively Individual points represent the mean abundance of all identified taxa at genus level. Red points indicate taxa with a statistically significant difference in abundance (greater than 2 log fold change) between till practices as determined by DeSEQ2 analysis. **B** and **D** represent differentially abundant taxa for slurry and digestate applied soils, respectively. In **B**, bars with a yellow outline highlight taxa found only where slurry had been applied, compared to no application. In (**D**), bars with a yellow outline highlight taxa found only where digestate had been applied, compared to no application. Light colour-filled bars represent the difference in mean abundance between slurry (**B**) and digestate (**D**) application, the darker coloured bars indicate the log2 fold change

DESeq2 was used to identify genera whose abundance was at least 2 log2-fold different following application of a specific amendment. Three analyses were run to analyse the differences in community structure between samples with one of the three soil amendments and no amendment samples. For slurry-applied soils, 4 bacterial genera were identified as significantly different (Fig. 4A) from samples where there had been no amendment. These genera were: bacteriap25 (phylum Myxococcota), *Frankiales* (Actinobacteriota), *Colimonas* (Proteobacteria), and *Zixibacteria* (Zixibacteria) (Fig. 4B). All were found to be upregulated with the application of slurry, except for bacteriap25, the mean abundance of which was found to be upregulated without amendment. For digestate-applied soils, 27 significantly differentially abundant taxa were identified at genus level (Fig. 4C), belonging to 12 phyla: Crenarchaerota, Myxococcota, Proteobacteria, Methylomirabilota, Desulfobacterota, Firmicutes, Acidobacteriota, Actinobacteriota, Chloroflexi, Armatimonadota, Cyanobacteria, and Halobacterota (Fig. 4D). Notably, of the 8 taxa upregulated where digestate had been applied, 7 belonged to the phylum Firmicutes. For soils where FYM had been applied, no taxa were found to be significantly different from samples where there had been no main soil amendment (data not shown). Considering βNTI, there were no significant differences between soil amendments (Supplementary Fig. S3A). In addition, the functional resilience of the communities was calculated by comparing functional and taxonomic distances. Higher levels of resilience were found where slurry and digestate had been applied, though under-representation of digestate samples should be taken into consideration (Supplementary Fig. S4A–C).

**Fig. 5:**
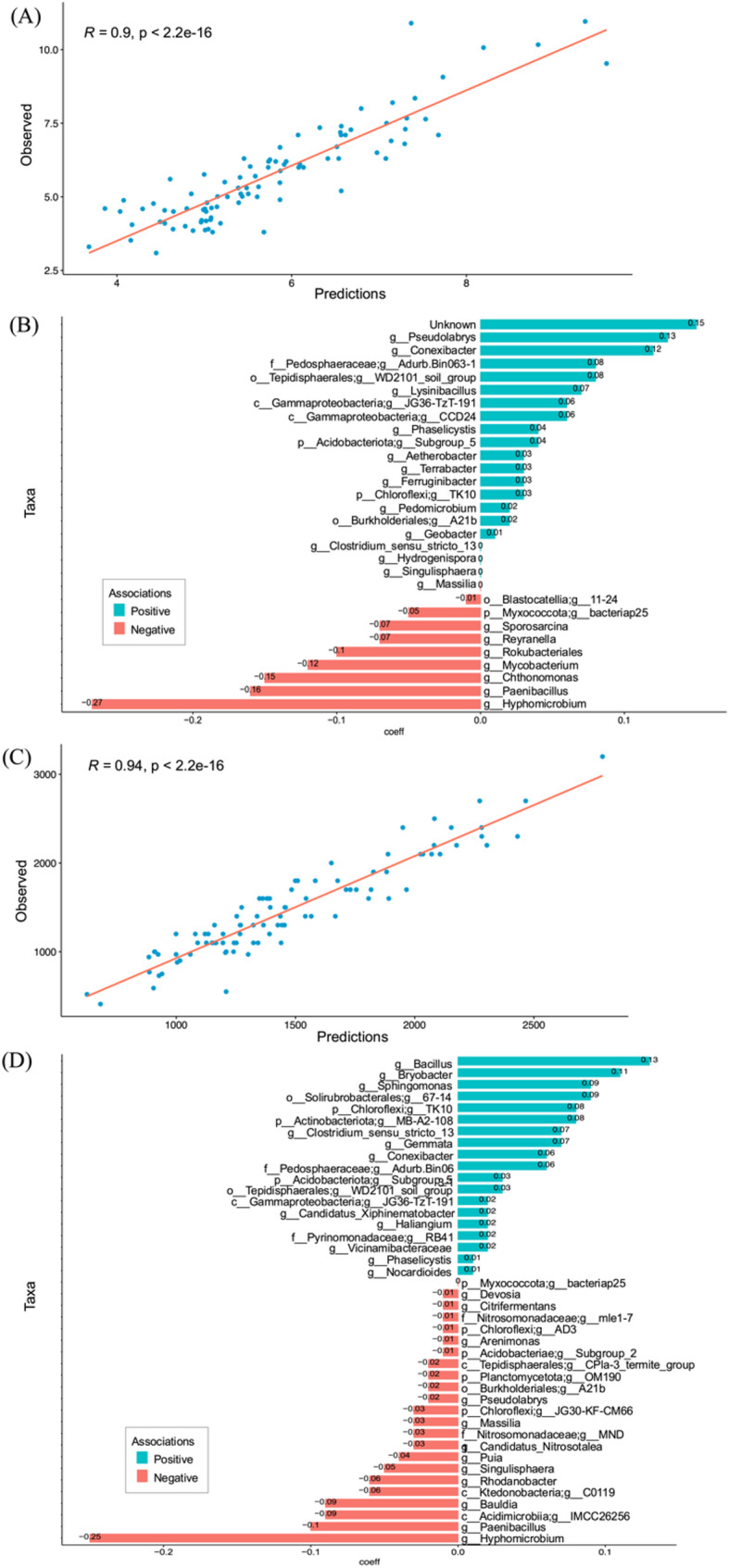
Results of the differential abundance using CODA-glmnet between application of a main soil amendment for LOI and soil calcium. **A** and **C** scatter plots visualise the model predictions through CODA-LASSO and actual values used in the CODA-LASSO regression (y-axis) such as LOI and soil calcium, respectively. **B** and **D** represent associations of taxa with LOI and soil calcium, respectively. Blue coloured bars represent bacterial species that are positively associated for LOI (**B**) or soil calcium (**D**), red bars represent bacterial species that are negatively associated under with soil calcium or LOI under application of any organic soil amendment

To assess the relative influence of environmental variables and management practices on the composition of the microbial communities, the CODA-LASSO algorithm was applied. Soil amendment application was divided into two groups (no soil amendment applied; any soil amendment applied). The results indicate association of certain taxa under application of soil amendments with respect to specific variables, with scatterplots to indicate the model fit (Figs. 5A and 4C). From the results of the RDA-PERMANOVA, we analysed the microbial relationships between the two continuous variables that were found to be significant using all three diversity metrics: LOI and soil calcium (*p* < 0.05) (Figs. 5B and 4D). For LOI, *Pseudolabrys*, *Conexibacter*, and *ADurb.Bin063-1* (family Pedosphaeraceae) were the three main genera found to be positively associated with soil amendment application (Fig. 5B), though taxonomically unassigned OTUs were most positively associated with increased levels of LOI. *Hyphomicrobium*, *Paenibacillus* and *Chthonomonas* were the main three genera negatively associated when organic soil amendment had been applied (Fig. 5B). Conversely, *Bacillus*, *Bryobacter*, and *Sphingomonas* were the main genera found to be more positively associated with soil calcium where organic amendment had been applied. The top three genera negatively associated with soil calcium levels where organic amendments had been applied were *Hyphomicrobium, Paenibacilus* and *IMCC26256* of class Acidimicrobiia (Fig. 5D).

Based on the results of the RDA-PERMANOVA analysis, a GLLVM was fitted using the following variables: soil calcium, crop two years before sampling, farm ID, and main soil amendment applied. *Bauldia*, *Mycobacterium*, Chloroflexi genus *AD3* and *Candidatus Nitrosotalea* were negatively associated with digestate application (Supplementary Fig. S6). *Candidatus Nitrosotalea* was strongly positively associated with FYM application but negatively associated with slurry application, compared to where no organic amendment had been applied. In addition, *Hydrogenispora*, *Devosia*, and *AD3* (phylum *Chloroflexi*) were found to be negatively associated with slurry application. PERMANOVA without the additional redundancy analysis identified soil texture, rotation type, magnesium, sodium and potassium as significant covariates that were not found to be significant after accounting for redundancy with other variables using forward/reverse selection (RDA). Variables including straw incorporation, cultivation method, nematicide application, and soil nutrient content (magnesium, sodium) were not significantly associated with microbial community structure (*p* < 0.05) (Table S6).

### Impact of soil texture on microbial communities

**Fig. 6:**
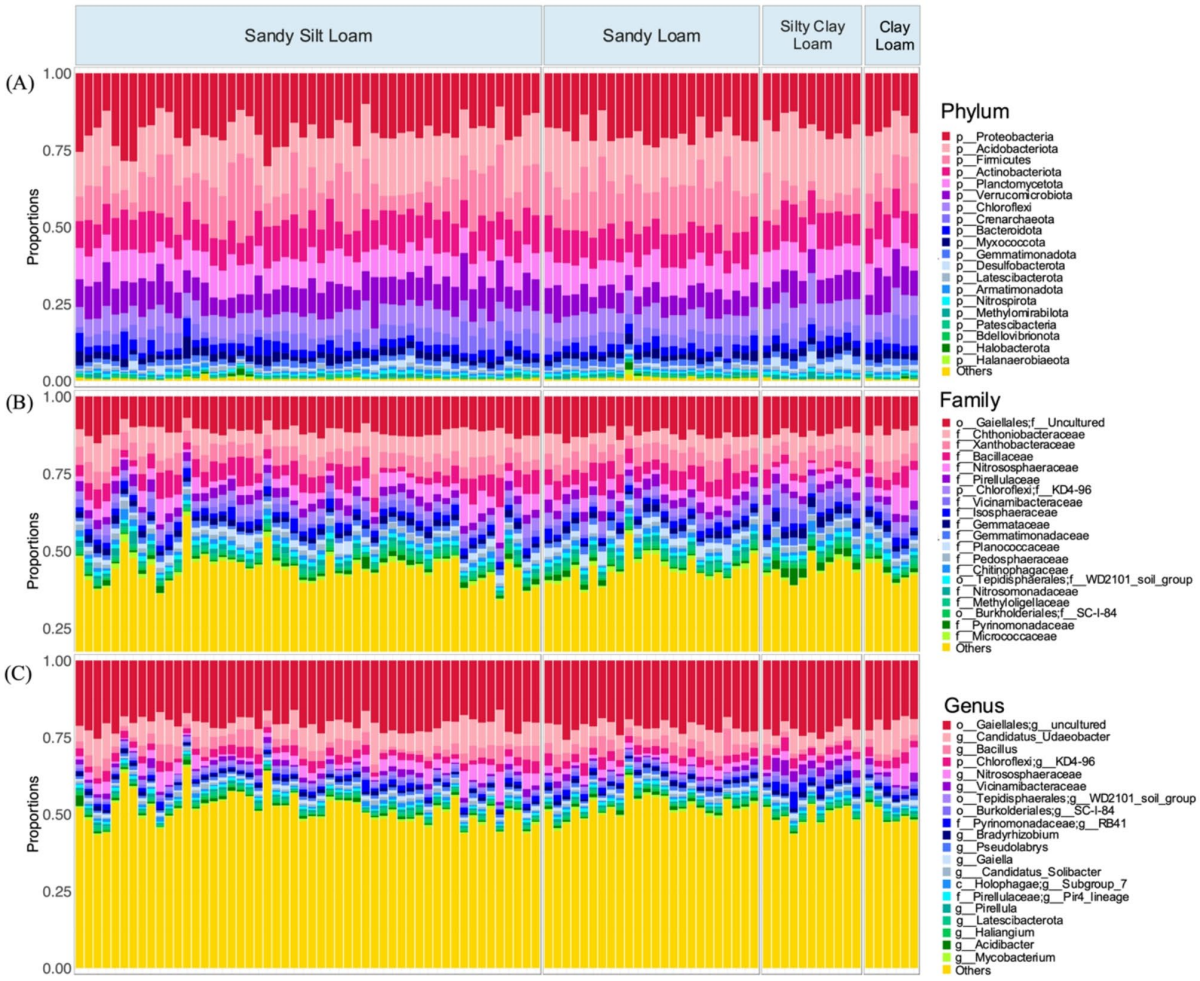
Proportional abundances of the 20 most abundant taxa at the resolution of phylum, family and genus across the four soil textures sampled

With respect to soil texture, silty clay loam had the lowest significant taxonomic (using Chao1’s measure of richness) and functional richness (Shannon’s measure of diversity), as compared to both sandy loam and sandy silt loam (Supplementary Fig. S2) for alpha diversity. Sandy loam soils had phylogenetically distinct bacterial communities from the other three soil textures, which exhibited some degree of overlap in both composition and phylogeny using Bray–Curtis and UniFrac measures of diversity respectively. Additionally, silty clay loam showed distinct separation using HMS (Supplementary Fig. S2B). Silty clay loam also had significantly reduced values for βNTI compared to the other soil textures (*p* < 0.01) (Supplementary Fig. S3B). By comparing functional and taxonomic distances, clay loam was found to have the greatest resilience, followed by silty clay loam. Sandy loam and sandy silt loam soils had similar, but lower levels of functional resilience to environmental perturbation (Supplementary Fig. S5). The community composition of samples split by soil texture can be seen in Fig. 6. At phyla level, sandy loam soils had relatively fewer Verrucomicrobiota and more Firmicutes than other soil textures, while silty clay loam type soils had fewer Firmicutes and more Acidobacteria. At family level, silty clay loam types had relatively fewer Bacilliaceae and more Pyrinomonadaceae. At increasing taxonomic resolution, a greater proportion of OTUs were classified as others for all soil textures.

### Core microbiome and its response to environmental drivers

**Fig. 7:**
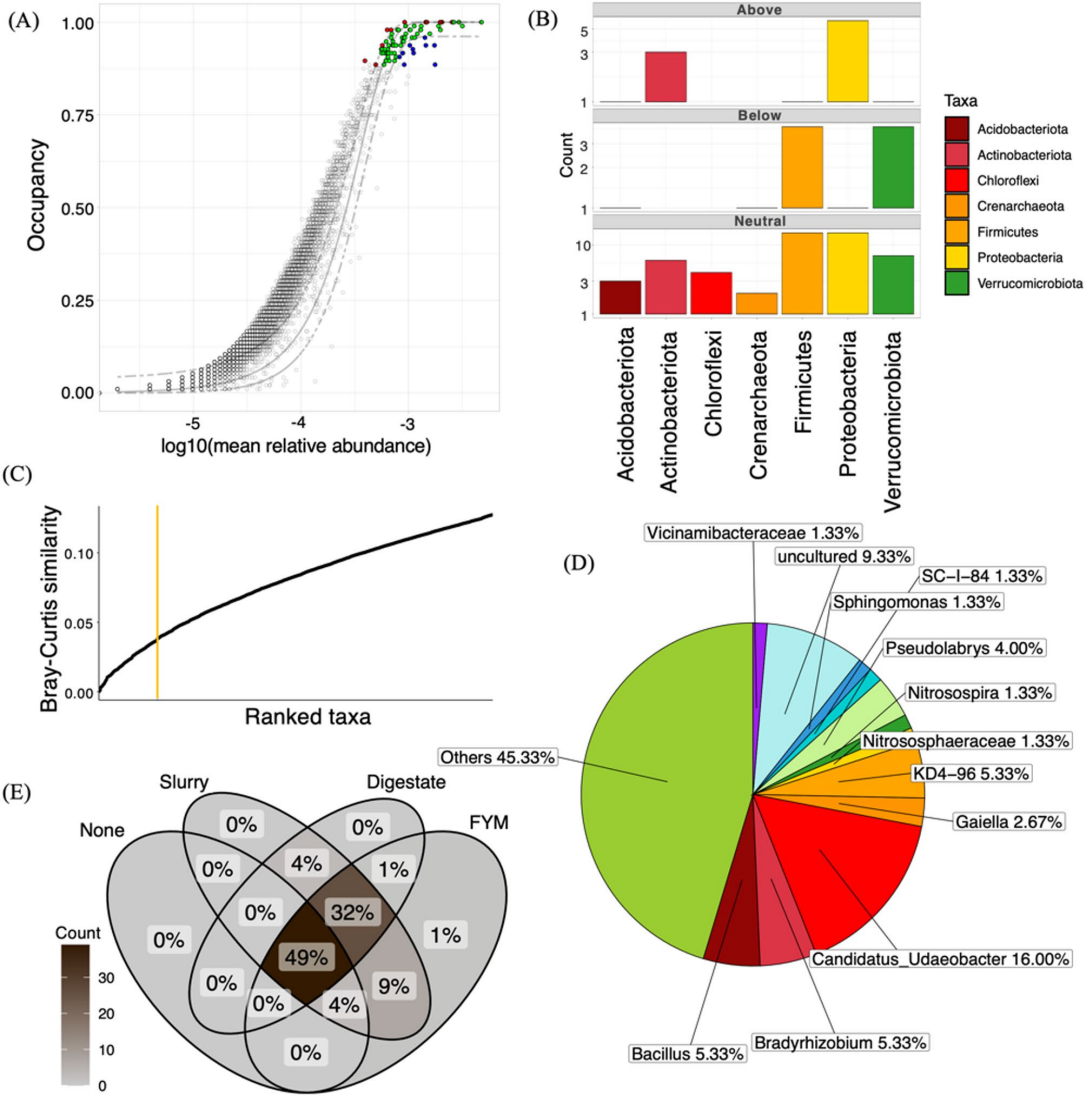
Core microbiome identified through a dynamic approach is represented by species occupancy abundance diagrams incorporating a site-specific occupancy criterion for a soil amendment occupancy model. **A** Points above the model are selected for by the environment and shown in red, points below the model are dispersal limited and shown in blue. The neutral core is shown in green colour. **B** Barplot representing the count of the number of OTUs fitted with the neutral model (Neutral; driven by stochastic processes), above the neutral model (Above; selected by the environment), and below the neutral model (Below; selected by dispersal limitation). **C** Ranked OTUs in terms of the Bray–Curtis contribution of OTUs. OTUs are added iteratively until the addition of one or more OTUs offers diminishing returns on the explanatory value of beta diversity. This threshold is represented by the vertical yellow line. **D** Core microbiome phylum composition for the occupancy model. **E** Venn diagram showing the overlap between 100% prevalent core OTUs (i.e., those OTUs observed in all samples) between different amendment types

The core microbiome based on organic amendment specific occupancy (digestate, farmyard manure (FYM), slurry, and no amendment) across replicates identified core taxa that had a minimum occupancy threshold of ~ 87.5% across all samples (Fig. 7A), suggesting a high degree of overlap of the core taxa between samples. Between the different occupancy models, there was a 49% sharing of unique core OTUs (Fig. 7D) including the occupancy model where no amendment was applied. Of the occupancy models where amendment had been applied, e.g., slurry, FYM and digestate, 32% of unique core OTUs were shared, with the highest pair-wise sharing of 9% unique core OTUs between slurry and FYM.

We found a total of 80 unique core OTUs that were fitted above the 95% confidence interval of the neutral model for the occupancy (Fig. 7A), the proportion of which can be seen in Fig. 7C. Among these, those that were fitted above the neutral model were dominated by *Bradyrhizobium*, *Bacillus*, and *Pir4* (family Pirelllaceae), OTUs of the families Bacillaceae, Micrococcaceae, Xanthobacteraceae, and OTUs of the order Bacillales. Of these, OTUs of Xanthobacteraceae and Bacillaceae were the most dominant. Those that were fitted below the model were dominated by *Nitrososophaeraceae*, *Pseudolabrys*, *Nitrosospira*, *Candidatus Udaeobacter*, *Bacillus*, and OTUs of the order Bacillales (see Supplementary Data Table S2). OTUs belonging to phylum Actinobacteriota and Proteobacteria were fitted above the neutral model (selected for by the environment), while OTUs belonging to the phyla Firmicutes and Verrucomicrobiota were fitted below the neutral model (dispersal limited) (Fig. 7B). The relative abundance of different phyla in the subset of core OTUs is shown in Fig. 7E. At phylum level, the core microbiome was dominated by Firmicutes, Proteobacteria and Verrucomicrobiota. The core microbial community was largely dominated by unclassified taxa at genus level, with *Candidatus Udaeobacter*, *Bradyrhizobium*, *Bacillus*, and *KD4*-*96* being the most abundant classified taxa (can also be seen in Fig. 6), while *Vicinamibacteraceae, Sphingomonas, SC-I-84 (*order *Burkholderiales), Nitrosospira,* and *Nitrososphaeraceae* were the least abundant taxa.

The taxonomic coverage of the core OTUs at different occupancies is shown in Supplementary Fig. S7. For digestate, the clades *Conexibacteraceae* and *Pirellulaceae* were found to be more abundant in comparison to other amendment types, and the clade *Terrabacter* was present (Supplementary Fig. S7A). For FYM, *Terrabacter* was absent, but both *Nitrosospira* and *SC-I-84* (order Burkholderiales) clades were present. The clade *Pirellulaceae* was present but not in as great abundance as in the digestate occupancy model (Fig. S7B). For slurry, the clades *Pirelllulaceae*, *Conexibacteraceae*, *Nitrospira*, *SC-I-84*, *Terrabacter, Nitrososphaeraceae,* and *Vicinamibacteraceae* were absent (Fig. S7C). This may be affected by the sample size. The occupancy model of no amendment contained all clades listed prior, except for the clade *Terrabacter* (Fig. S7D). *Terrabacter* was also absent in the collated occupancy results (Fig. S7E).

## Discussion

### Introduction

Our findings demonstrate that both soil texture and organic amendment application significantly influences the composition and diversity of the soil microbiome. The variation in microbial community structure found within this study extends our understanding of microbiomes in agroecosystems and the effect agricultural management can have on both the taxonomic and functional diversity of soil microorganisms [53].

To consider ecological structuring mechanisms, βNTI values indicated the highest level of phylogenetic dispersal occurred where digestate had been applied and the lowest where slurry had been applied. This suggests that stronger selective pressures are imposed by slurry application and that stochastic events may play a more significant role in shaping microbial community structure in digestate-applied soils. This is reflected in the higher number of differentially abundant taxa found in soils amended with digestate. We hypothesise that this contrast is driven by differences in amendment properties: Slurry application may impose strong, short-term selective pressures due to high localised concentrations of labile organic matter and ammonium, leading to transient anoxia and selection of tolerant taxa [16]. Conversely, digestate tends to promote greater structural complexity by increasing pore size variability and enhancing aggregate formation [54]. This enhanced physical heterogeneity reduces the uniformity of environmental filtering and increases the contribution of dispersal and stochastic process in community assembly [24].

However, a degree of caution should be applied given the comparatively low sample size for this amendment. Additionally, soil texture was also found to significantly alter microbial community composition, therefore the effectiveness of organic amendments in shaping microbial communities may depend on the interplay between the amendment type and the inherent characteristics of the soil texture [55]. Understanding the interaction between environmental conditions and organic amendments in shaping microbial community composition is a crucial aspect for targeted agricultural practices to cultivate healthy and resilient soils. These findings have practical implications for developing sustainable agricultural practices, in particular the selection of amendments for taxonomic and phylogenetically diverse soil communities, and to optimise soil health [56].

### Impact of soil texture and amendments on microbial diversity and community structure

The inherent temporal and spatial variation in physical and chemical properties of soils across microbiome studies creates a challenge for assessing the long-term impacts of soil management practices [57]. Soil texture significantly influences diversity metrics because microbial community assembly is impacted by the physicochemical factors that change with texture, e.g., pore size, nutrient holding capacity, and water holding capacity [9, 24, 58]. In the present study, sandy loam and silty clay loam soils had the highest and lowest taxonomic richness respectively, which is consistent with the idea that larger pore spaces found in sandy loam soils can accommodate a wider variety of microbial life compared to finer texture soils [11, 59]. While sandy loam soils may harbour a wider variety of microbial species, their lower nutrient-holding capacity and less diverse microhabitats may limit the functional diversity compared to clay loam soils [60]. Clay loam soils exhibited the highest functional diversity; clay minerals provide adhesion sites for microbes and the unique physical structure of clay-based soils, with variations in moisture, oxygen, and nutrient availability, can facilitate the establishment of a diverse range of microhabitats, capable of supporting a wide range of functional groups [61].

Chau et al. [24] reported that an increased number of isolated water films led to increased species richness in coarser soils, while soil texture was considered a secondary driver (after pH) in shaping the soil microbial community. Both soil pH and soil texture can be impacted by the addition of organic amendments which introduce organic matter into soils, altering the structure of soil and its ability to retain moisture [9]. The addition of different forms of organic amendments led to varying levels of organic matter across soils adding a layer of variability to the factors structuring microbial communities in the present study.

Where soil amendments were added, microbial diversity significantly increased following the application of slurry (taxonomic diversity) and digestate (functional diversity), which aligns with previous findings that found increased diversity following the introduction of external microbial communities through organic amendments [21]. The addition of digestate resulted in the greatest number of differentially abundant taxa with many of these taxa associated with low oxygen environments e.g., *Methylomirabilota*, [62] and *Halobacterota*, [63]; breakdown of organic matter, e.g., *Armatimonadota* [64]; or soils with high C to N ratios, e.g., *Crenarchaerota* [65]. The influx of nutrient resources from slurry and digestate application may also have stimulated existing populations, promoting niche differentiation and potentially favouring less abundant or dormant groups, resulting in higher levels of diversity [19]. This is supported by the significant increase in functional diversity following digestate application, suggesting the establishment of microbes with a broader range of functional capabilities in alignment with the breakdown processes during anaerobic digestion [18, 66]. However, a degree of caution should be observed given the comparatively low sample size for both digestate and slurry samples.

Our findings also showed a positive association between soil calcium and organic matter content with higher levels of microbial diversity in sandy loam soils and where digestate had been applied. While pH is considered a key environmental factor shaping microbial communities, edaphic factors including soil mineral composition also have variable effects on soil microbiome community structure [8, 67, 68]. This is evident in the present study, where calcium appears to be a stronger predictor than pH. Calcium influences soil processes through various mechanisms including pH regulation, nutrient availability and soil structure, suggesting that the specific edaphic context can modulate the relative importance of different environmental factors. Consequently, studies such as Cho et al. [69] have found that pH was not the only factor affecting microbial community structure.

In addition to the role calcium plays in modulating soil physio-chemical conditions, calcium can have an active role in the growth and function of microorganisms, especially involving surface-adhesion and for biofilm-forming bacteria; in this way, calcium is an important cofactor involved in soil organic carbon cycling [70]. In this work, Shabtai et al. showed that increased soil calcium levels promoted the enrichment of specific phyla, namely the phyla Bacillota and Actinomycetota, and affected the formation of organo-mineral associations, which was found to directly impact community function. Furthermore, work by Neal and Glendining [71] highlighted the importance of soil calcium by demonstrating that exchangeable calcium is a primary driver of phosphohydrolase gene abundance, a gene essential in microbial phosphorus acquisition, encoding an enzyme responsible for hydrolysing organic phosphate compounds into bioavailable inorganic phosphate. These findings support the hypothesis that calcium is a major edaphic factor in shaping microbial communities. This is particularly relevant when considering the observed positive correlation between increased calcium levels and both functional and taxonomic diversity.

### Differentially abundant taxa and environmental drivers

To further explore the environmental drivers of microbial community structure, we employed a Generalised Linear Latent Variable Model (GLLVM). Chloroflexi genus *AD3* was negatively associated with both slurry and digestate application, consistent with their preference for oligotrophic conditions. *Candidatus Nitrosotalea*, positively associated with FYM application but negatively with digestate, may benefit from the diverse microbial community in manure and may be outcompeted by anaerobic bacteria in digestate. The upregulation of *Candidatus Nitrosotalea* following FYM application suggests increased soil acidity, as this ammonia-oxidising archaea is often found in acidic environments [72]. This could contribute to increased soil fertility through ammonia oxidation.

Using DeSEQ2, we identified taxa whose abundance was found to be significantly influenced by different treatments. The upregulation of Firmicutes following digestate application aligns with their adaptation to nutrient-rich, anaerobic environments. Firmicutes, many of which are endospore-forming, thermophilic and filamentous, are well-suited to environments with high organic matter content and complex soil structures. These bacteria can act as keystone taxa, playing a critical role in maintaining the overall function and balance of the soil ecosystem by contributing to nutrient cycling, plant growth promotion and building healthy soil [73–75]. This taxon helps to maintain a healthy microbiome by inhibiting the growth of pathogens and contributes to the formation of soil aggregates, improving soil structure and water retention capacity. In contrast, Chloroflexi genus *AD3*, adapted to oligotrophic conditions, may have been outcompeted by other taxa under nutrient-rich conditions [76]. The lack of significant changes in community composition following application of farmyard manure suggests that the microbial community found in the manure was similar to that of the soil, or that the introduced taxa were outcompeted by native species. Alternatively, the soil environment may have been inhospitable to the FYM’s microbial community. It should be noted that the sample size presents a challenge in fully interpreting these results. Overall, the range of analysis suggests an interaction between organic amendment and soil texture with the relative balance of deterministic and stochastic processes varying between treatment types and soil textures.

### The core microbiome

In addition to the differences in microbial diversity found between organic amendments and soil texture, our analysis was able to identify 80 core taxa that were consistently present across all samples, highlighting a shared microbial guild. Methods for identification of the core microbiome are numerous and use a variety of methods, such as searching for OTU enrichment within communities [77], using network topology data and identifying core network taxa [78], the identification of dominant phylotypes [79], and the abundance-occupancy distribution model, which was used in this study [48]. Despite the differences in approach, the unified objective was to identify a core set of taxa to better understand the microbial community structure and its role in ecological processes. Using abundance-occupancy models to identify the core taxa may offer new directions for targeted interventions to optimise soil health and ecosystem services [79]. Here, the core microbiome was dominated by taxa such as *Bradyrhizobium*, *Bacillus*, and *Pirellulaceae*, suggesting a stable section of the soil community that is resilient to variations in organic amendment application and exists across farms and soil textures. The neutral model revealed certain core taxa, belonging to Actinobacteriota (e.g., *Bradyrhizobium*, many species of which are nitrogen fixers [80]) and Proteobacteria (e.g., *Pirellulaceae*, anaerobic ammonia-oxidizing bacteria [81]), were selected for by environmental factors, while other taxa, belonging to Firmicutes and Verrucomicrobiota, were more dispersal limited. This suggests that as with the wider soil community, a combination of ecological processes shapes the assembly of the core microbiome and that taxa belonging to specific phyla respond to environmental pressures in different ways.

We observed variations in the relative abundance of specific taxa within the core microbiome across different occupancies. The presence of the *Conexibacteraceae* clade in digestate-treated soils suggests potential decreases in soil respiration as this clade is often associated with slow-growing bacteria that exhibit lower metabolic activity [82]. However, this clade also contains known denitrifiers, which while not directly contributing to carbon-based respiration, may influence overall soil respiration dynamics by altering electron acceptor availability [83]. Conversely, the presence of the *Terrabacter* clade, associated with nitrogen fixation, organic matter decomposition and plant growth promotion, is a positive indicator of soil health [84]. These findings highlight that while a largely conserved core microbiome exists across all samples, specific organic amendments can still selectively influence its composition, impacting both taxonomy and function.

### Conclusion

In conclusion, this work highlights the importance of understanding the interplay between agronomic management and soil texture, and the importance of these factors in collectively shaping microbial communities. By understanding these interactions, we can advance regenerative agricultural practices that promote soil health, nutrient cycling and carbon sequestration [85, 86]. The observed changes in microbial community structure have implications for soil health; enrichment of Actinobacteria and Proteobacteria can improve soil structure, enhance soil organic matter content and facilitate nutrient cycling [64, 87]. This study identified key drivers of soil microbial diversity and community assembly, provided valuable insights for developing sustainable agricultural practices, and highlighted the importance of understanding these factors in the context of tailored management strategies for optimised microbial activity and ecosystem services. This study used state-of-the-art statistical analyses for in-depth insights into the impacts of soil amendments and texture on the soil microbiome. While our study incorporated rigorous statistical methods to mitigate any potential analytical biases, future studies may address certain limitations, such as the reliance on predicted metabolic profiles from PiCRUST2. Additionally, larger sample sizes and the investigation of underexplored factors like spatial autocorrelation combined with other modalities such as metagenomics or quantitative PCR could yield a more comprehensive understanding of the factors shaping the soil microbiome, including the often-overlooked trophic relationships with microbial predators (e.g. protozoa and nematodes) – a fundamental weakness of the vast majority of 16srRNA studies. By integrating advanced molecular techniques and statistical modelling, we can continue to unravel the complex interactions between soil, microbes and the environment. This research contributes to a deeper understanding of the ecological processes underpinning sustainable agriculture and informs the development of targeted strategies to cultivate healthier soil, providing a scientific basis for developing adaptation strategies to mitigate climate change and ensure food security.

## Supplementary Information

Below is the link to the electronic supplementary material.

Supplementary file1 (DOCX 1206 kb)

Supplementary file2 (CSV 25 kb)

Supplementary file3 (CSV 12 kb)

## Data Availability

The dataset supporting the conclusions of this article is available from the sequence read archive (SRA) database under Bioproject Submission PRJEB79745) https://www.ncbi.nlm.nih.gov/bioproject/PRJEB79745. In addition, metadata associated with the samples is provided in Supplementary\_Data\_Table\_S1.csv.

## Code availability

Some of the code used to generate the results are part of microbiomeSeq (https://github.com/umerijaz/microbiomeSeq), whilst other codes are available at http://userweb.eng.gla.ac.uk/umer.ijaz#bioinformatics.
