# Supplementary material for: From farm to microbe: organic amendments and soil texture as drivers of soil Microbiome composition
Source: Environ Microbiome. 2025 Dec 29;20:158. doi: 10.1186/s40793-025-00815-1 (PMC12752172; doi:10.1186/s40793-025-00815-1)
Supplement: Supplementary file 1 — Supplementary file1 (DOCX 1206 kb) [file 40793_2025_815_MOESM1_ESM.docx]

**Supplementary Materials**

**From farm to microbe: Organic amendments and soil texture as drivers of soil microbiome composition**

Maya Subberwal^1^, Madeline Giles^2#^, Roy Neilson^2^, David Roberts^2^, Sandra Caul^2^, Susan Mitchell^2^, and Umer Zeeshan Ijaz^1#^.

^1^ James Watt School of Engineering, University of Glasgow, UK

^2^ The James Hutton Institute, Invergowrie, Dundee DD2 5DA, UK

^#^ Both authors jointly directed this work and are co-corresponding authors: [maddy.giles@hutton.ac.uk](mailto:maddy.giles@hutton.ac.uk) and [Umer.Ijaz@glasgow.ac.uk](mailto:Umer.Ijaz@glasgow.ac.uk)

Table S1. Environmental conditions for all samples. Categorical data (represented with ‘(n)’) with all possible outcomes and the frequency of occurrence are shown here.

| Soil Texture | Date | Season (n) | Conventional/Organic (n) |
| --- | --- | --- | --- |
| Sandy Silt Loam | 17/03/2016-03/05/2016 | Spring:52 | Conventional:52 |
| Sandy Loam | 17/03/2016-03/05/2016 | Spring:24 | Conventional:24 |
| Silty Clay Loam | 21/02/2016-25/04/2016 | Spring:11 | Conventional:11 |
| Clay Loam | 21/03/2016-02/04/2016 | Spring:6 | Conventional:6 |

Table S2. Crop planting history. Description is similar to that of Supplementary Table S1.

| Soil Texture | **Crop at Sampling (n)** | **Crop planted 1 year before sampling (n)** | **Crop planted 2 years before sampling (n)** | **Crop planted 3 years before sampling (n)** | **Crop planted 4 years before sampling (n)** |
| --- | --- | --- | --- | --- | --- |
| Sandy Silt Loam | WW: 52 | SO: 7  SPEAS: 6  SPOT: 8  WOSR: 20  WW: 11 | FALW: 1  SB: 11  SO: 4  WB: 12  WO: 4  WOSR: 4  WPOT: 4  WW: 12 | CALA: 3  SB: 20  WB: 6  WOSR: 4  WPOT: 3  WW: 16 | CALA: 3  SB: 11  SO: 2  SPOT: 2  SWS: 4  WB: 6  WOSR: 4  WPOT: 12  WW: 8 |
| Sandy Loam | WW:24 | WOSR:16  WPOT:8 | SB:13  WB:8  WO:3 | SB:16  WPOT:1  WW:7 | SB:12  SPOT:2  WOSR:3  WPOT:6  WW:1 |
| Silty Clay Loam | WW:11 | SPEAS:3  WOSR:4  WW:4 | FALW:3  SO:3  WB:1  WOSR:4 | FALW:4  WB:1  WOSR:3  WW:3 | WB:4  WW:7 |
| Clay Loam | WW:6 | SO:1  SPEAS:3  WOSR:1  WW:1 | SO:1  WB:1  WW:4 | CALA:1  WB:1  WOSR:1  WW:3 | CALA:1  SB:1  SO:2  WB:2 |

Table S3. Mineral analysis. IQR stands for Inter Quartile Range.

| Soil Texture | **pH** | | **Phosphorus** | | **Potassium** | | **Magnesium** | | **Calcium** | | **Sodium** | | **LOI** | | **Moisture** | | **Temperature** | |
| --- | --- | --- | --- | --- | --- | --- | --- | --- | --- | --- | --- | --- | --- | --- | --- | --- | --- | --- |
|  | Mean | IQR | Mean | IQR | Mean | IQR | Mean | IQR | Mean | IQR | Mean | IQR | Mean | IQR | Mean | IQR | Mean | IQR |
| Sandy Silt Loam | 6.01 | 5.90-6.10 | 8.75 | 6.90-9.96 | 153.80 | 112.80-177.50 | 177.4 | 123.5-224.2 | 1457 | 1100-1700 | 15.64 | 11.90-17.23 | 5.67 | 4.60-6.30 | 314.3 | 288.0-334.0 | 8.38 | 8.00-9.00 |
| Sandy Loam | 5.93 | 5.70-6.10 | 11.93 | 7.70-15.62 | 137.10 | 97.33-171.50 | 127.4 | 96.0-151.2 | 1148 | 970-1225 | 11.20 | 7.50-12.65 | 5.15 | 4.15-5.51 | 295.1 | 284.0-316.5 | 8.0 | 6.0-9.5 |
| Silty Clay Loam | 6.23 | 6.10-6.30 | 5.67 | 4.15-6.70 | 193.50 | 171.00-209.00 | 191.8 | 169.5-213.0 | 2082 | 1900-2250 | 16.62 | 14.20-16.10 | 7.23 | 6.65-7.75 | 292.5 | 289.8-295.2 | NA | NA |
| Clay Loam | 5.97 | 5.78-6.00 | 7.84 | 3.88-11.93 | 157.80 | 130.5-183.8 | 228.2 | 164.5-277.8 | 1917 | 1725-2175 | 19.3 | 15.6-21.1 | 6.61 | 6.07-7.05 | 346.2 | 323.8-372.0 | NA | NA |

Table S4. Management conditions. Description is similar to that of Supplementary Table S1.

| Soil Texture | **Tillage (n)** | **Straw incorporation (n)** | **Main soil amendment (n)** | **Second soil amendment (n)** | **Cultivation method (n)** | **Nematicide applied (n)** |
| --- | --- | --- | --- | --- | --- | --- |
| Sandy Silt Loam | Conventional:49  Reduced:3 | Removed:24  Incorporated:28 | Digestate:3  FYM:11  None:36  Slurry:2 | FYM:5  None:47 | Mouldboard:51  Minimum tillage:1 | No:44  Yes:8 |
| Sandy Loam | Conventional:24 | Removed:12  Incorporated:12 | Digestate:1  FYM:3  None:20 | FYM:1  None:23 | Mouldboard:24 | No:13  Yes:11 |
| Silty Clay Loam | Conventional:1  Reduced:10 | Removed:1  Incorporated:10 | None:10  Slurry:1 | FYM:1  None:10 | Mouldboard:8  Minimum tillage:3 | No:11 |
| Clay Loam | Conventional:3  Reduced:3 | Removed:3  Incorporated:3 | FMY:2  None:3  Slurry:1 | FYM:1  None:5 | Mouldboard:6 | No:6 |

Table S5. Redundancy analysis with both (forward/reverse) selection was performed to select the most important environmental variables that explain variation in the community matrices. The initial set of variables considered are as follows (with those selected in the final PERMANOVA models in bold case): **Farm ID**, Soil Texture (*Sandy Silt Loam, Sandy Loam, Silty Clay Loam, Clay Loam*), **Crop Year 4** (*WW, CALA, SB, SO, SPOT, SWS, WB, WOSR, WPOT*), **Crop Year 3** (*WW, CALA, FALW, SB, WB, WOSR, WPOT*), **Crop Year 2** (*WW, FALW, SB, SO, WB, WO, WOSR, WPOT*), **Crop Year 1** (*WW, SO, SPEAS, SPOT, WOSR, WPOT*), **Rotation Type** (*Combinable only, Combinable and Roots including Potatoes*), **Straw Incorporation** (*Incorporated, Baled and removed*), Main Soil Amendment (*None, Digestate, FYM, Slurry*), **Second Soil Amendment** (*None, FYM*), **Cultivation Method** (*Minimum Tillage, Mouldboard Ploughing*), Nematicide Applied (*Yes, No*), **Soil pH**, **Soil Phosphorus**, Soil Potassium, Soil Magnesium, **Soil Calcium**, Soil Sodium, **Soil LOI**. Here, Df, SS, R^2^, F and p are Degrees of Freedom, Sum of Squared Error, Coefficient of Determination, F-statistic, and p-value, respectively.

|  | Covariates | Df | SS | R^2^ | F | p |  |
| --- | --- | --- | --- | --- | --- | --- | --- |
| Bray-Curtis Distance |  |  |  |  |  |  |  |
|  | Calcium | 1 | 1.2067 | 0.0538 | 5.3796 | 0.001 | *** |
|  | Second soil amendment | 1 | 0.6068 | 0.02705 | 2.7051 | 0.001 | *** |
|  | Cultivation method | 1 | 0.5101 | 0.02274 | 2.274 | 0.002 | ** |
|  | pH | 1 | 0.4858 | 0.02166 | 2.1657 | 0.002 | ** |
|  | Crop year 1 | 5 | 1.5631 | 0.06969 | 1.3937 | 0.001 | *** |
|  | Crop year 4 | 8 | 2.4878 | 0.11092 | 1.3863 | 0.001 | *** |
|  | LOI | 1 | 0.3471 | 0.01548 | 1.5475 | 0.022 | * |
|  | Farm ID | 4 | 1.0374 | 0.04625 | 1.1562 | 0.064 | . |
|  | Crop year 2 | 1 | 0.2391 | 0.01066 | 1.0658 | 0.287 |  |
|  | Phosphorus | 1 | 0.2615 | 0.01166 | 1.166 | 0.142 |  |
|  | Residuals | 61 | 13.6829 |  | 0.61008 |  |  |
|  | Total | 85 | 22.4283 |  |  |  |  |
| Unweighted UniFrac |  |  |  |  |  |  |  |
|  | Calcium | 1 | 1.0768 | 0.03807 | 3.5569 | 0.001 | *** |
|  | Second soil amendment | 1 | 0.5578 | 0.01972 | 1.8427 | 0.001 | *** |
|  | Cultivation method | 1 | 0.5099 | 0.01803 | 1.6843 | 0.001 | *** |
|  | pH | 1 | 0.5014 | 0.01773 | 1.6564 | 0.001 | *** |
|  | Crop year 1 | 5 | 1.884 | 0.06662 | 1.2447 | 0.001 | *** |
|  | Straw incorporation | 1 | 0.4144 | 0.01465 | 1.3689 | 0.002 | ** |
|  | Crop year 3 | 6 | 2.1185 | 0.07491 | 1.1663 | 0.001 | *** |
|  | Farm ID | 4 | 1.5056 | 0.05324 | 1.2433 | 0.001 | *** |
|  | LOI | 1 | 0.3379 | 0.01195 | 1.1161 | 0.112 |  |
|  | Residuals | 64 | 19.3747 |  | 0.68508 |  |  |
|  | Total | 85 | 28.281 |  |  |  |  |
| Hierarchical Meta-Storms | |  |  |  |  |  |  |
|  | Farm ID | 12 | 0.0085708 | 0.37244 | 4.3598 | 0.001 | *** |
|  | Calcium | 1 | 0.0010558 | 0.04588 | 6.4448 | 0.002 | ** |
|  | Crop year 3 | 5 | 0.0020722 | 0.09005 | 2.5298 | 0.001 | *** |
|  | pH | 1 | 0.0003652 | 0.01587 | 2.2294 | 0.045 | * |
|  | Phosphorus | 1 | 0.0002998 | 0.01303 | 1.8302 | 0.119 |  |
|  | Residuals | 65 | 0.0106485 |  | 0.46273 |  |  |
|  | Total | 85 | 0.0230124 |  |  |  |  |
| Significance codes: ‘.’ p<0.1, ‘*’ p<0.05, ‘**’ p<0.01, ‘***’ p<0.001 | | | | | | | |

Table S6. PERMANOVA analysis was performed to select the most important environmental variables that explain variation in the community matrices. The initial set of variables considered are as follows (with those selected in the final PERMANOVA models in bold case): **Farm ID**, Soil Texture (*Sandy Silt Loam, Sandy Loam, Silty Clay Loam, Clay Loam*), **Crop Year 4** (*WW, CALA, SB, SO, SPOT, SWS, WB, WOSR, WPOT*), **Crop Year 3** (*WW, CALA, FALW, SB, WB, WOSR, WPOT*), **Crop Year 2** (*WW, FALW, SB, SO, WB, WO, WOSR, WPOT*), **Crop Year 1** (*WW, SO, SPEAS, SPOT, WOSR, WPOT*), **Rotation Type** (*Combinable only, Combinable and Roots including Potatoes*), **Straw Incorporation** (*Incorporated, Baled and removed*), Main Soil Amendment (*None, Digestate, FYM, Slurry*), **Second Soil Amendment** (*None, FYM*), **Cultivation Method** (*Minimum Tillage, Mouldboard Ploughing*), Nematicide Applied (*Yes, No*), **Soil pH**, **Soil Phosphorus**, Soil Potassium, Soil Magnesium, **Soil Calcium**, Soil Sodium, **Soil LOI**. Here, Df, SS, R^2^, F and p are Degrees of Freedom, Sum of Squared Error, Coefficient of Determination, F-statistic, and p-value, respectively.

|  | **Covariates** | **Df** | **SS** | **R^2^** | **F** | **p** |  |
| --- | --- | --- | --- | --- | --- | --- | --- |
| Bray-Curtis Distance |  |  |  |  |  |  |  |
|  | Farm ID | 12 | 5.2233 | 0.23289 | 1.94189 | 0.001 | *** |
|  | Soil texture | 3 | 0.7784 | 0.0347 | 1.1575 | 0.102 |  |
|  | Crop year 4 | 5 | 1.4738 | 0.06571 | 1.31501 | 0.002 | ** |
|  | Crop year 3 | 2 | 0.5433 | 0.02422 | 1.21182 | 0.081 | . |
|  | Rotation type | 1 | 0.2552 | 0.01138 | 1.13852 | 0.197 |  |
|  | pH | 1 | 0.3646 | 0.01625 | 1.6264 | 0.009 | ** |
|  | Phosphorus | 1 | 0.2774 | 0.01237 | 1.23743 | 0.099 | . |
|  | Potassium | 1 | 0.2118 | 0.00944 | 0.94506 | 0.539 |  |
|  | Magnesium | 1 | 0.2457 | 0.01096 | 1.09623 | 0.228 |  |
|  | Calcium | 1 | 0.2408 | 0.01074 | 1.07418 | 0.283 |  |
|  | Sodium | 1 | 0.2094 | 0.00934 | 0.93407 | 0.53 |  |
|  | LOI | 1 | 0.2766 | 0.01233 | 1.23388 | 0.106 |  |
|  | Residuals | 55 | 12.3282 |  | 0.54967 |  |  |
|  | Total | 85 | 22.4283 |  |  |  |  |
| Unweighted UniFrac |  |  |  |  |  |  |  |
|  | Farm ID | 12 | 5.56 | 0.1966 | 1.53718 | 0.001 | *** |
|  | Soil texture | 3 | 1.0209 | 0.0361 | 1.12895 | 0.023 | * |
|  | Crop year 4 | 5 | 1.7941 | 0.06344 | 1.19045 | 0.001 | *** |
|  | Crop year 3 | 2 | 0.682 | 0.02412 | 1.13138 | 0.05 | * |
|  | Rotation type | 1 | 0.3306 | 0.01169 | 1.09679 | 0.133 |  |
|  | pH | 1 | 0.4233 | 0.01497 | 1.40452 | 0.001 | *** |
|  | Phosphorus | 1 | 0.3551 | 0.01256 | 1.17816 | 0.054 | . |
|  | Potassium | 1 | 0.2925 | 0.01034 | 0.97029 | 0.567 |  |
|  | Magnesium | 1 | 0.3132 | 0.01107 | 1.03895 | 0.284 |  |
|  | Calcium | 1 | 0.307 | 0.01086 | 1.01855 | 0.331 |  |
|  | Sodium | 1 | 0.2875 | 0.01017 | 0.95392 | 0.636 |  |
|  | LOI | 1 | 0.3366 | 0.0119 | 1.11677 | 0.115 |  |
|  | Residuals | 55 | 16.5781 |  | 0.58619 |  |  |
|  | Total | 85 | 28.281 |  |  |  |  |
| Hierarchical Meta-Storms |  |  |  |  |  |  |  |
|  | Farm ID | 12 | 0.0085708 | 0.37244 | 4.3801 | 0.001 | *** |
|  | Soil texture | 3 | 0.0007228 | 0.03141 | 1.4775 | 0.116 |  |
|  | Crop year 4 | 5 | 0.0016575 | 0.07203 | 2.033 | 0.009 | ** |
|  | Crop year 3 | 2 | 0.0006662 | 0.02895 | 2.0429 | 0.036 | * |
|  | Rotation type | 1 | 0.0001934 | 0.00841 | 1.1862 | 0.284 |  |
|  | pH | 1 | 0.0005105 | 0.02218 | 3.1306 | 0.008 | ** |
|  | Phosphorus | 1 | 0.0004816 | 0.02093 | 2.9535 | 0.014 | * |
|  | Potassium | 1 | 0.0001329 | 0.00577 | 0.8149 | 0.496 |  |
|  | Magnesium | 1 | 0.0002702 | 0.01174 | 1.6568 | 0.14 |  |
|  | Calcium | 1 | 0.000418 | 0.01817 | 2.5636 | 0.032 | * |
|  | Sodium | 1 | 0.0002628 | 0.01142 | 1.6114 | 0.136 |  |
|  | LOI | 1 | 0.0001572 | 0.00683 | 0.9639 | 0.431 |  |
|  | Residuals | 55 | 0.0089685 |  | 0.38972 |  |  |
|  | Total | 85 | 0.0230124 |  |  |  |  |
| Significance codes: ‘.’ p<0.1, ‘*’ p<0.05, ‘**’ p<0.01, ‘***’ p<0.001 | | | | | | | |


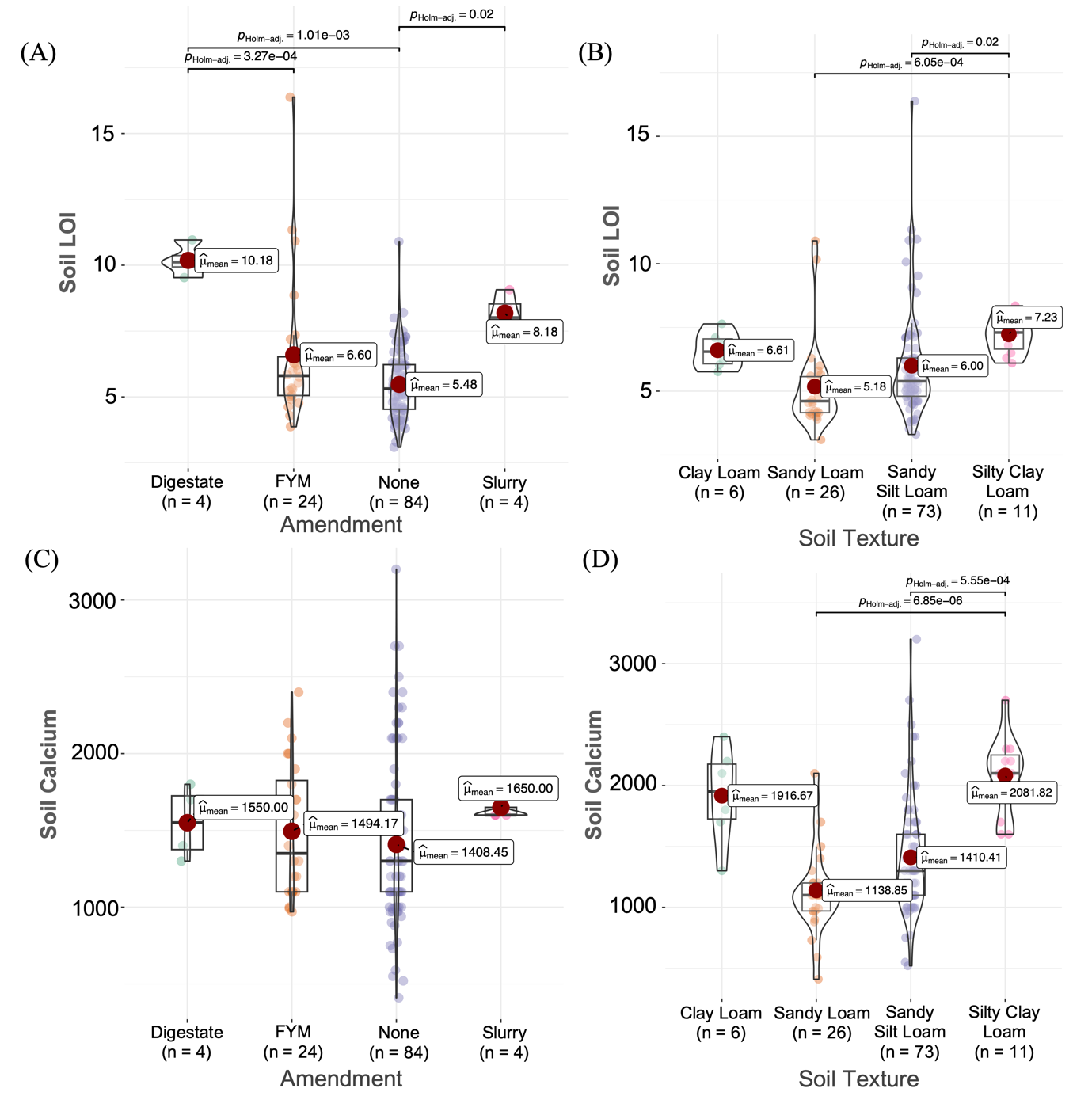


Figure S1. Violin plots detailing variation in soil LOI and soil calcium. (A) and (B) show variation in soil LOI between application of different amendments, and soil texture respectively. (C) and (D) shows variation in calcium respectively for different amendments and soil texture. The horizontal lines at the top of the plots denote different levels of significance (p<0.05). The mean value is represented by the red circle in the centre of the boxplots.

(B)


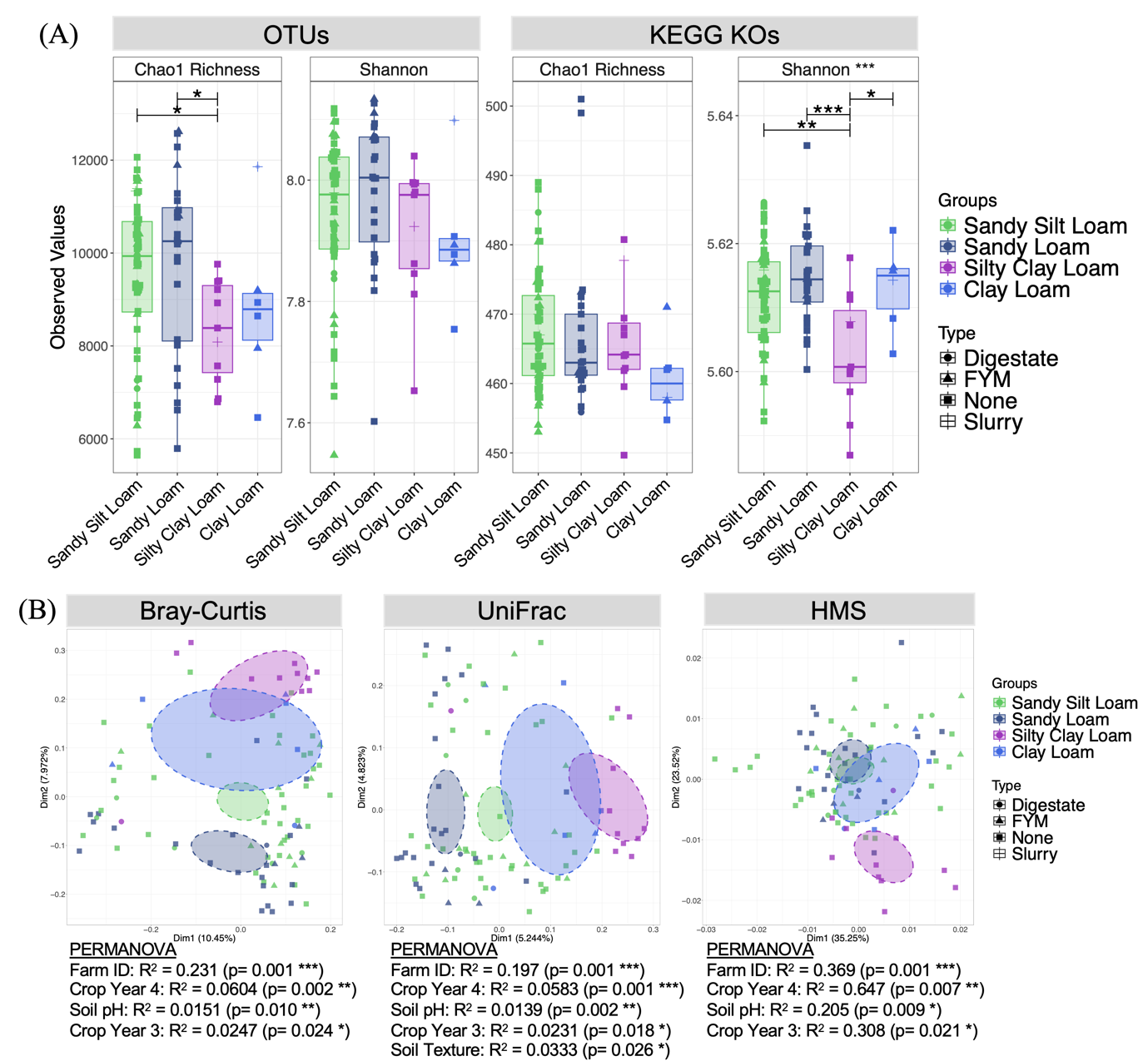
Figure S2. (A) shows boxplots representing alpha diversity across soil textures showing Chao1 measure of richness and Shannon entropy for OTUs (left panel) and KEGG orthologs (right panel). Lines connect two categories where the differences were significant (ANOVA) with **p* < 0.05, ***p* < 0.01, or ****p* < 0.001. (B) shows PCoA plots representing beta diversity with PERMANOVA statistics below. The shape of points represents different soil amendments.


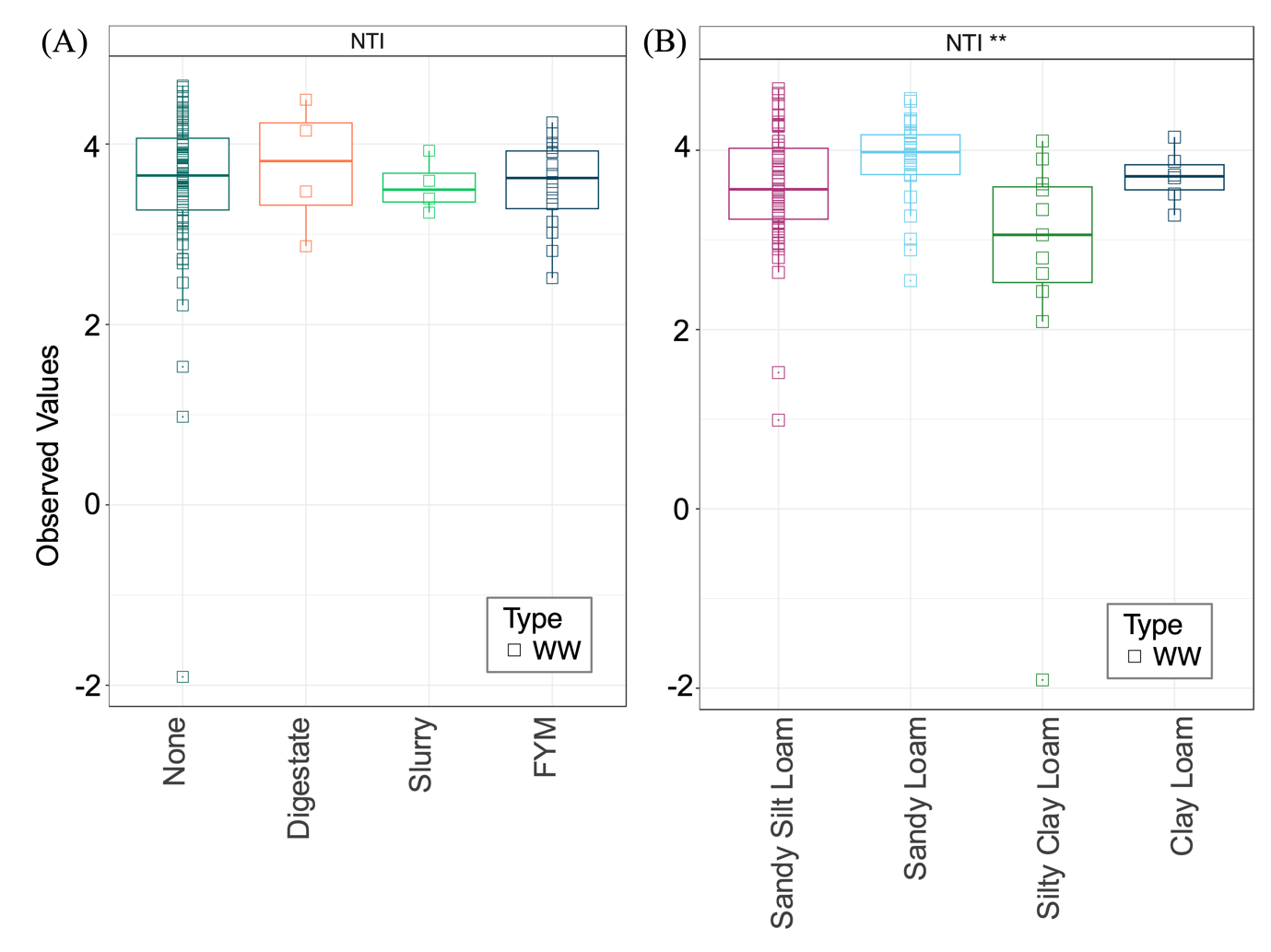


Figure S3. Nearest taxon index (NTI) of microbial communities derived from agricultural soils with (A) different soil amendments applied, and (B) different soil textures. Higher NTI values indicate a phylogenetically dispersed microbial community i.e., increased role of environment in influencing the microbial community assemblage. The strip title also shows the result from ANOVA if the differences are statistically significant: **p* < 0.05, ***p* < 0.01, or ****p* < 0.001.


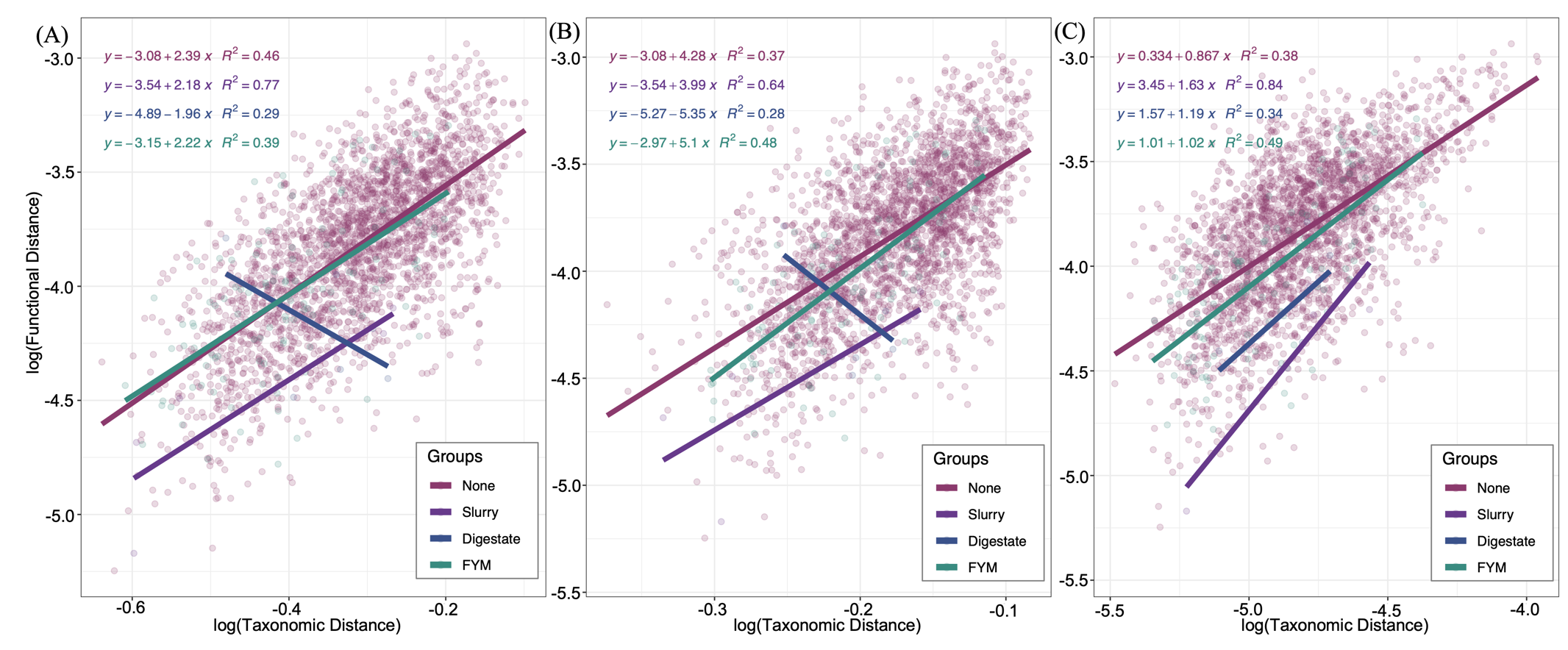
Figure S4. Comparison of robustness of soil microbial communities for different amendments. Regression lines are fitted through distances between any two samples (shown as a dot) with x-axis showing taxonomic perturbation between any two samples, whilst the y-axis shows the corresponding functional perturbation. The slope of the line then serves as a proxy of resilience (robustness), e.g., a smaller angle between the fitted lines and the x-axis suggest that significantly more taxonomic shift is required to cause a substantial shift in the function, i.e. the group of samples is more resilient to perturbation. Three different taxonomic distances matrices were used; (A) Bray-Curtis, (B) Unweighted UniFrac, and (C) Weighted Unifrac. The functional distance between the samples was calculated using Hierachical Meta-Storms. Note that the lines should have a positive slope (The regression lines for “Digestate” group is a negative results possibly due to undersampling).


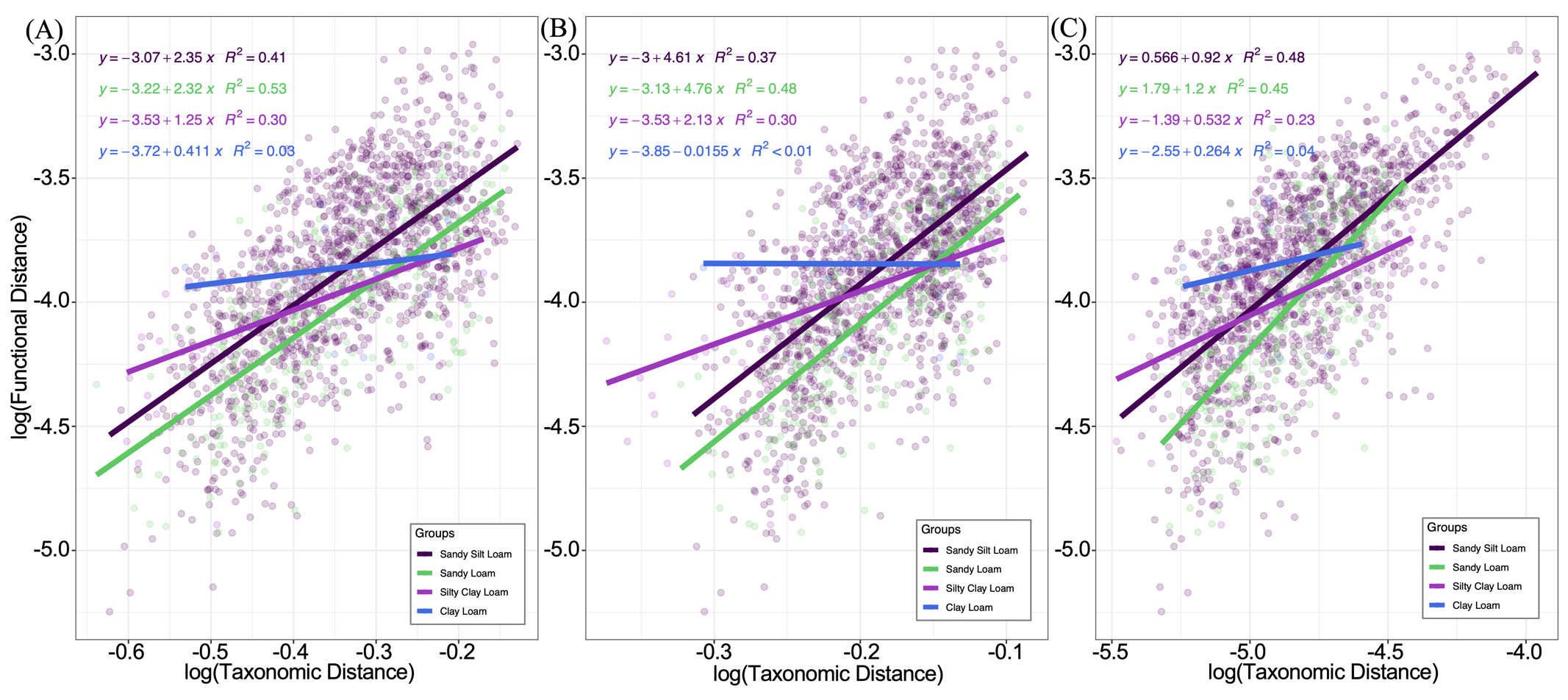
Figure S5. Comparison of robustness of soil microbial communities for different soil types. The description of the plot is similar to what is provided in the legend of Figure S4.


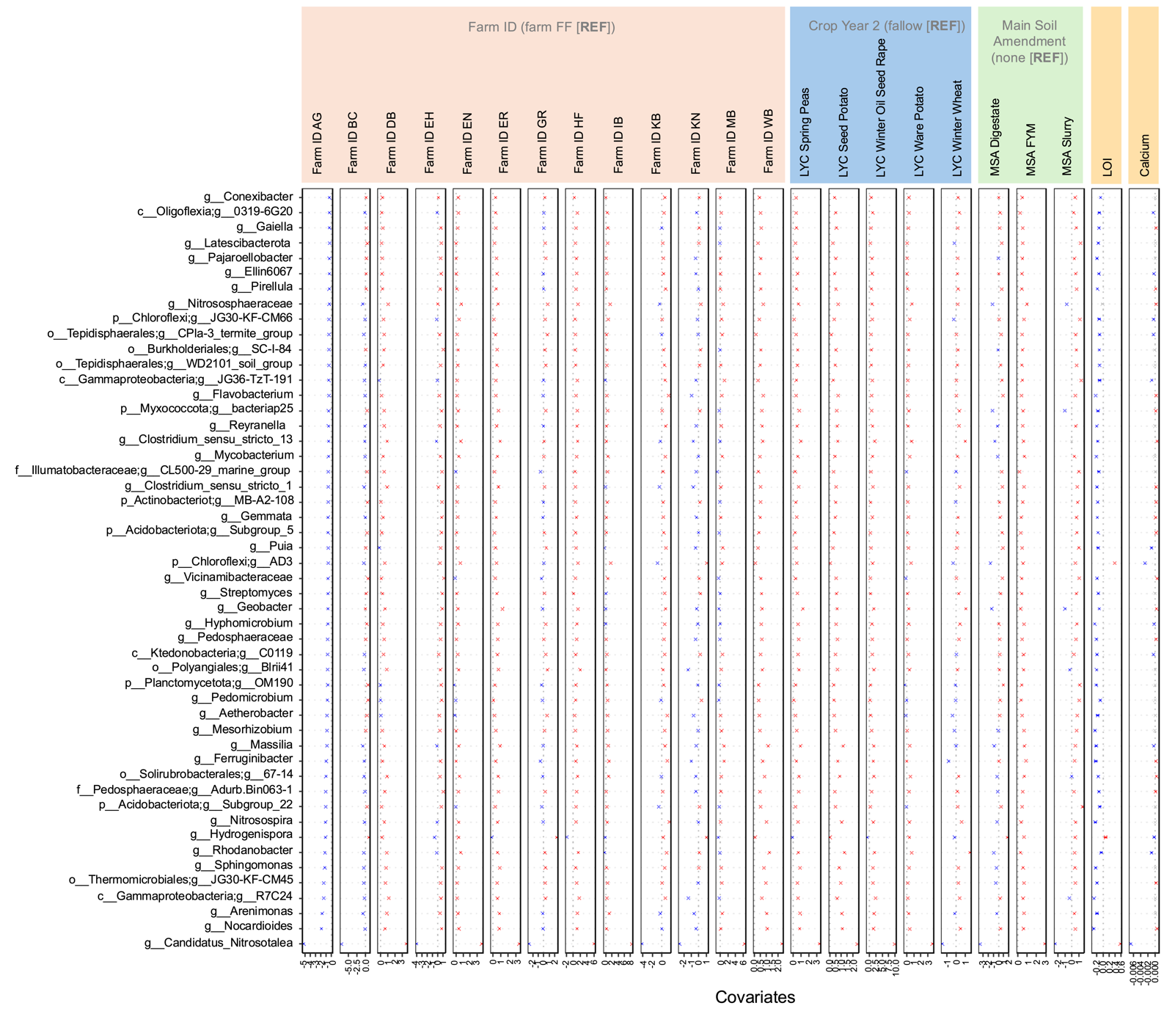


Figure S6. β-coefficients recovered for different covariates using GLLVM procedure after collating OTU abundances at genus level and retaining top-100 most abundant genera. For continuous variables, such as *LOI*, and *Calcium*, red and blue crosses represent a positively and negatively associated relationship between a particular genus and these variables. For categorical variables, the positive and negative associations are interpreted with respect to the reference variable. These are shown on the top: “fallow” for *Crop Year 2* [fallow]; “FF” for *Farm ID*; and “none” amendment for *Main Soil Amendment*, respectively. As an example, a red cross for *Digestate* under *Main Soil Amendment* for g__Adurb.Bin063-1 implies an increase in its abundance when compared to no amendment. Grey crosses represent a statistically insignificant relationship where the 95% confidence interval of the β-coefficients cross the 0 boundary. Where the genera are well explored in the literature, and their roles are known, they are further annotated with the symbols with the key given on the top left. The x axis represents the beta coefficient values for each covariate. Continued on next page.


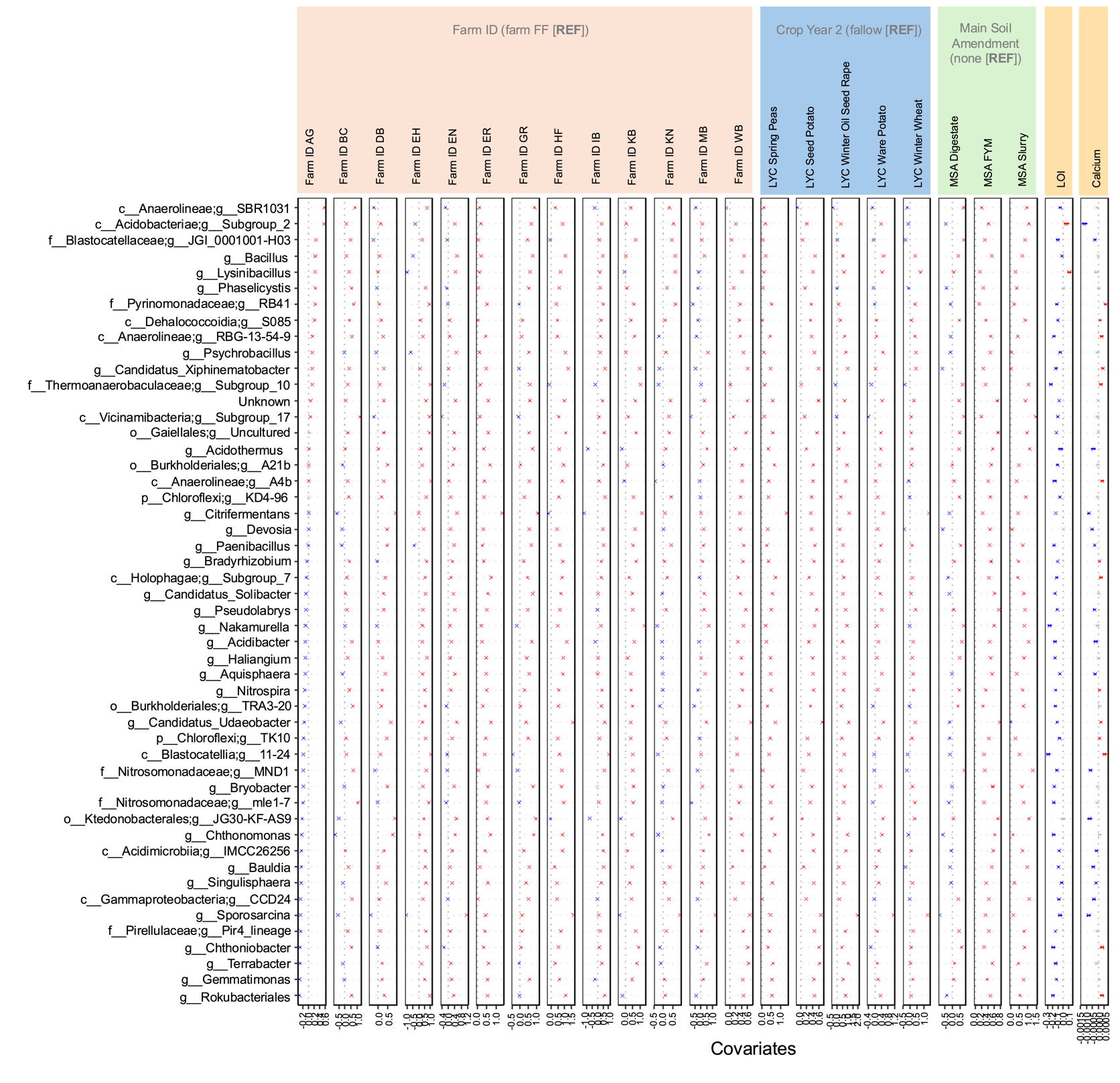
Figure S6. Cont.


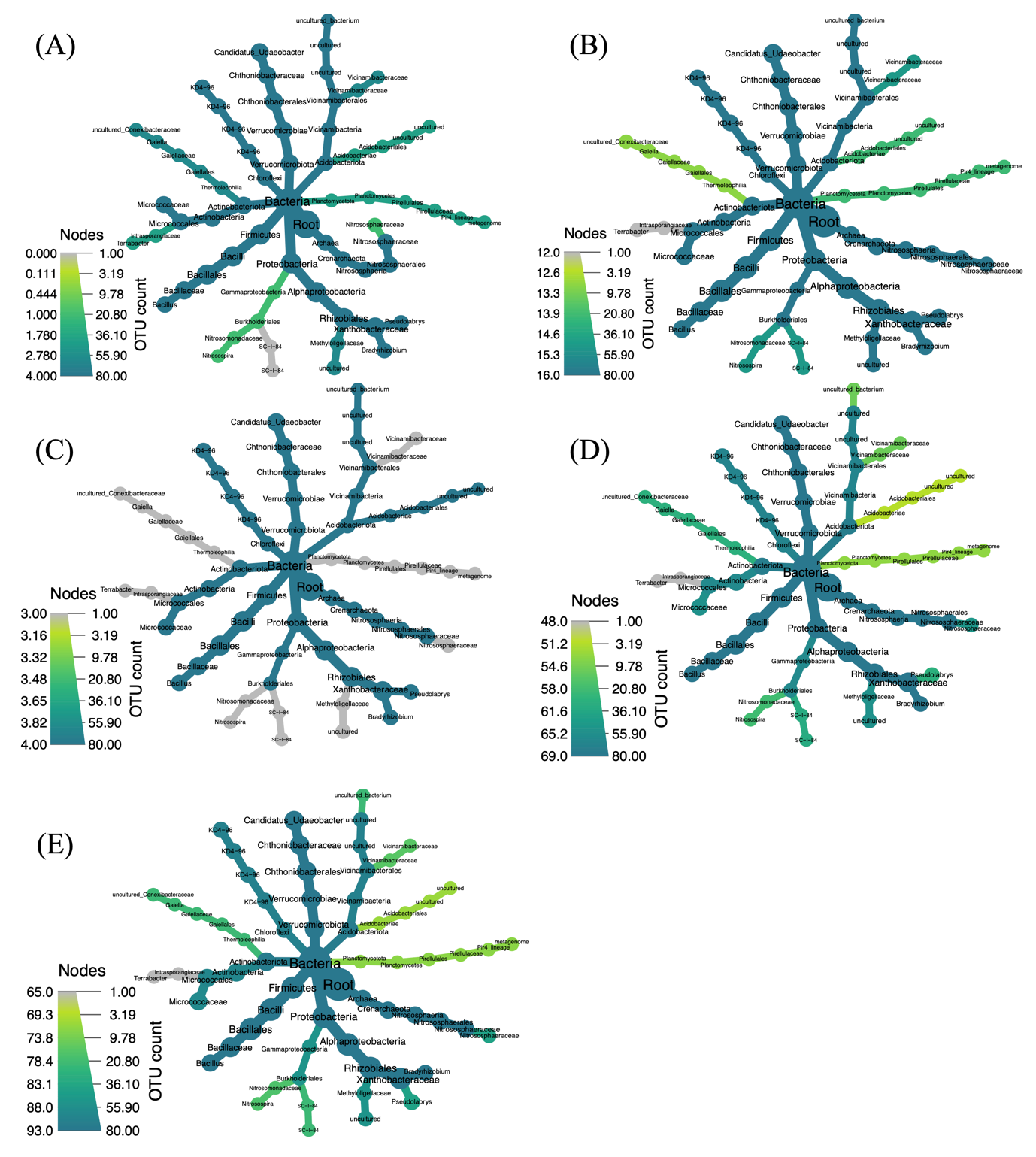
Figure S7. Taxonomy tree of the abundances of core microbiome identified using the dynamic core microbiome inference strategy using different occupancy models. Darker shades represent higher abundances whilst the lighter shades indicate where lineages were in low abundance. The size of the nodes corresponds to the number of unique taxa. The core microbiome for different occupancies are shown in (A) digestate, (B) farmyard manure (FYM), (C) slurry, and (D) no amendment, whilst (E) shows the collated results i.e., (A), (B), (C), and (D) all added together.
